# Therapeutic Potential Effect of Glycogen Synthase Kinase 3 Beta (GSK-3β) Inhibitors in Parkinson Disease: Exploring an Overlooked Avenue

**DOI:** 10.1007/s12035-024-04003-z

**Published:** 2024-02-17

**Authors:** Areej Turkistani, Hayder M. Al-kuraishy, Ali I. Al-Gareeb, Ali K. Albuhadily, Athanasios Alexiou, Marios Papadakis, Mohamed M. Elfiky, Hebatallah M. Saad, Gaber El-Saber Batiha

**Affiliations:** 1 Department of Pharmacology and Toxicology, College of Medicine, Taif University, 21944 Taif, Saudi Arabia; 2 Department of Clinical Pharmacology and Medicine, College of Medicine, Mustansiriyah University, P.O. Box 14132, Baghdad, Iraq; 3 University Centre for Research & Development, Chandigarh University, Chandigarh-Ludhiana Highway, Mohali, Punjab India; 4 Department of Research & Development, Funogen, Athens Greece; 5 Department of Research & Development, AFNP Med, 1030 Vienna, Austria; 6 Department of Science and Engineering, Novel Global Community Educational Foundation, Hebersham, NSW 2770 Australia; 7 Department of Surgery II, University Hospital Witten-Herdecke, Heusnerstrasse 40, University of Witten-Herdecke, 42283 Wuppertal, Germany; 8 Anatomy Department, General Medicine Practice Program, Batterjee Medical College, Jeddah, Saudi Arabia; 9 Anatomy Department, Faculty of Medicine, Menoufia University, Shibin El Kom, Al Minufya Egypt; 10 Department of Pathology, Faculty of Veterinary Medicine, Matrouh University, Matrouh, 51744 Egypt; 11 Department of Pharmacology and Therapeutics, Faculty of Veterinary Medicine, Damanhour University, Damanhour, 22511 AlBeheira Egypt

**Keywords:** Parkinson’s disease, GSK-3β

## Abstract

Parkinson’s disease (PD) is a progressive neurodegenerative disease of the brain due to degeneration of dopaminergic neurons in the substantia nigra (SN). Glycogen synthase kinase 3 beta (GSK-3β) is implicated in the pathogenesis of PD. Therefore, the purpose of the present review was to revise the mechanistic role of GSK-3β in PD neuropathology, and how GSK-3β inhibitors affect PD neuropathology. GSK-3 is a conserved threonine/serine kinase protein that is intricate in the regulation of cellular anabolic and catabolic pathways by modulating glycogen synthase. Over-expression of GSK-3β is also interconnected with the development of different neurodegenerative diseases. However, the underlying mechanism of GSK-3β in PD neuropathology is not fully clarified. Over-expression of GSK-3β induces the development of PD by triggering mitochondrial dysfunction and oxidative stress in the dopaminergic neurons of the SN. NF-κB and NLRP3 inflammasome are activated in response to dysregulated GSK-3β in PD leading to progressive neuronal injury. Higher expression of GSK-3β in the early stages of PD neuropathology might contribute to the reduction of neuroprotective brain-derived neurotrophic factor (BDNF). Thus, GSK-3β inhibitors may be effective in PD by reducing inflammatory and oxidative stress disorders which are associated with degeneration of dopaminergic in the SN.

## Introduction

Parkinson’s disease (PD) is a chronic and progressive neurodegenerative disease of the brain [1]. PD is characterized by motor symptoms including tremors, rigidity, and bradykinesia, and non-motor such as dementia, cognitive dysfunction, sleep disorders, and depression that may develop by decades before motor symptoms [1, 2]. Aging is the main factor that predisposes the development of PD and is linked with its severity. PD affects 1–3% of the general population aged more than 60 years. However, PD that may be developed below the age of 50 years is known as an early-onset PD, though onset of PD below 21 years is called juvenile PD. PD prevalence is more common in men than women that might due to higher levels of neuroprotective estrogen [3, 4]. The pathogenesis of PD is related to the progressive degeneration of dopaminergic neurons in the substantia nigra (SN) and the accumulation of Lewy bodies in the survival neurons. Lewy bodies are mainly formed by the deposition of α-Syn which is also found in other neurological disorders called synucleinopathies. Of note, loss of 70% of dopaminergic neurons in the SN is developed before the development of PD symptoms [3, 4].

The presence and contribution of α-Syn to PD is controversial and might be pathogenic or a compensatory increased to reduce dopaminergic neuronal loss. Two types of PD are identified, idiopathic (sporadic) PD which form 90% of cases whereas familial PD account for 10% only [5, 6]. Mutation of α-Syn is associated with the development of familial PD [7].

**Fig. 1:**
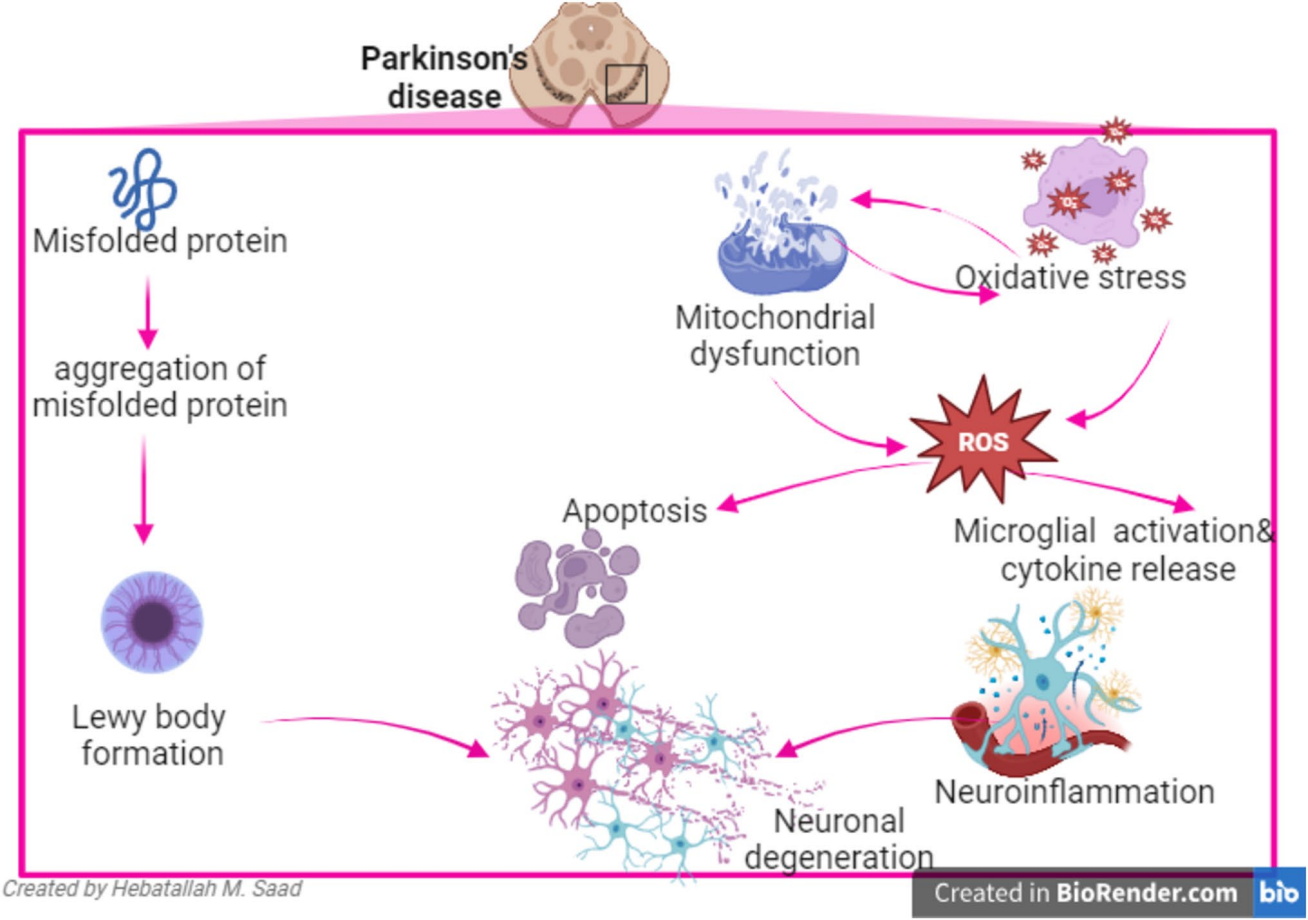
Pathophysiology of PD: induction formation of misfolded proteins by different causes promote aggregation of misfolded proteins and formation of Lewy body which induce neuronal degeneration. In addition, development of mitochondrial dysfunction and oxidative by different causative factors implicated in the pathogenesis lead to generation of reactive oxygen species (ROS) which cause direct neuronal apoptosis or indirectly through activation of microglia and the development of neuroinflammation lead to neuronal degeneration

It has been reported that genetic alterations in PD are found since early embryonic life that predispose to the development of PD after the age of 60 years [7]. Genetic alteration can interrelate with different environmental factors in the pathogenesis of PD [8]. It has been hypothesized that three temporal phases including triggers (like environmental toxins), facilitators (like peripheral inflammation), and aggravators (like autophagy dysfunction) are required for the pathogenesis of PD [9]. For example, gut dysbiosis and alteration of the nasal microbiome promote the deposition of α-Syn and the development of non-motor symptoms of PD [9]. Systemic inflammation in chronic metabolic disease facilitates neuroinflammation and degeneration of dopaminergic neurons SN with the accumulation of α-Syn [10]. Defective autophagy which acts as an aggravator promotes PD neuropathology by reducing the clearance of α-Syn [9]. Furthermore, mitochondrial dysfunction, oxidative stress, apoptosis, and dysfunction of growth factors contribute to the pathogenesis of PD [11] (Fig. 1).

It has been shown that glycogen synthase kinase 3 beta (GSK-3β) is intricate in the pathogenesis of PD [12]. Though, the underlying mechanism of GSK-3β in PD neuropathology is not fully clarified. Therefore, the objective of the present review was to revise the mechanistic role of GSK-3β in PD neuropathology. In addition, we try to revise the potential therapeutic role of GSK-3β inhibitors in PD.

## GSK-3β and Neurodegenerative Disorders

GSK-3 is a conserved threonine/serine kinase protein that regulates cellular anabolic and catabolic pathways by modulating glycogen synthase in response to biological stimuli [13]. In particular, GSK-3 is involved in neurodevelopment and synaptic plasticity, though GSK-3 is implicated in the development of neurodegeneration, cognitive dysfunction and bipolar disorders [14]. Two isozymes of GSK-3 including GSK-3α and GSK-3β are identified; they have 98% similarity with overlapping function [13].

Normally, GSK-3β is expressed in all brain regions; however, GSK-3α is expressed in specific brain regions such as the cerebral cortex, hippocampus, and Purkinje cells [15]. Signaling pathways involved with GSK-3β are mainly phosphoinositol 3 phosphatase kinase (PI3K) and Wnt/β-catenin [16]. GSK-3β regulates cell cycle signaling, cell proliferation, and DNA repair [17]. In addition, GSK-3β regulates cellular oxidative stress through modulation of the expression of nuclear factor erythroid 2-related factor 2 (Nrf2) [18]. Findings from preclinical and clinical studies illustrated that exaggerated GSK-3β activity is involved in progressive neurodegeneration in different neurodegenerative diseases [19–21].

### Alzheimer’s Disease

Alzheimer’s disease (AD) is the most common neurodegenerative disease characterized by progressive memory loss and cognitive impairment [22]. AD is developed due to progressive deposition of extracellular amyloid beta (Aβ) protein and intracellular neurofibrillary tangles (NFTs) which are formed by hyperphosphorylation of tau protein [23]. Accumulated Aβ in AD induces upregulation of GSK-3β which increases the activation of amyloid precursor protein (APP) leading to the generation of Aβ in a vicious cycle. Therefore, GSK-3β activity is augmented in AD leading to synaptic failure and impairment of synaptic plasticity leading to cognitive decline [19]. Moreover, GSK-3β promotes tau protein phosphorylation-induced neurodegeneration and increases AD neuropathology.

**Fig. 2:**
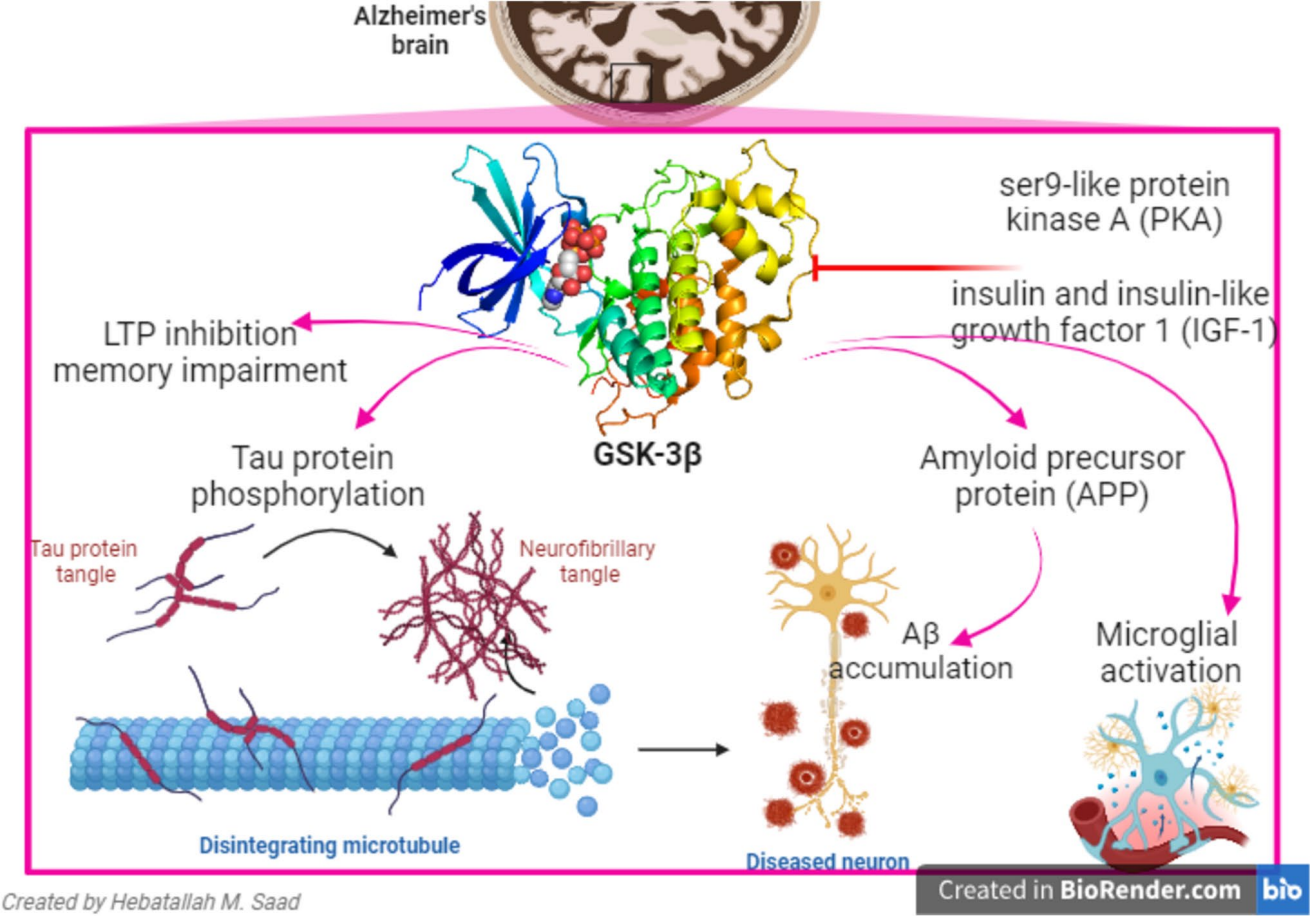
Role of GSK-3β in Alzheimer disease (AD): GSK-3β has an important role in the pathogenesis of AD by inducing amyloid precursor protein (APP) processing for Aβ and activation of microglia. GSK-3β by increasing tau protein phosphorylation causes memory impairment and inhibition of long-term potentiation (LTP). GSK-3β is inhibited by ser9-like protein kinase A (PKA) and insulin-like growth factor 1 (IGF-1)

However, the underlying mechanism for the overactivation of GSK-3β is remaining unidentified [19, 24]. Normally, GSK-3β activity is negatively inhibited by phosphorylation on ser9-like protein kinase A (PKA) by insulin and insulin and insulin-like growth factor 1 (IGF-1). Dysregulation of this pathway as in insulin resistance (IR) induces overexpression of GSK-3β [19, 25]. Brain IR in diabetes is associated with activation of GSK-3β due to failure of insulin and IGF-1 signaling [25]. A cohort study that involved AD patients showed that the active form of GSK-3β was increased in the frontal cortex neurons in the early stages of AD patients before accumulation of NFTs [24]. GSK-3β activation is energetic by phosphorylation of tau protein which results in disturbance of neuronal synaptic activity and the formation of neuronal plaques. Though the accumulation of Aβ plaques and intracellular NFTs has been well recognized as neuropathological hallmarks of the disease, the molecular mechanism has not been elucidated [24]. It has been shown that ginsenoside improves cognitive function by regulating oxidative stress, apoptosis, and neuroinflammation in experimental AD by inhibiting GSK-3β [26]. Likewise, tolfenamic acid constrains GSK-3β-mediated tau hyperphosphorylation in AD models [27]. This finding suggests that GSK-3β could be a primary event in the development of AD (Fig. 2). Therefore, inhibition of exaggerated GSK-3β could be effective against AD neuropathology.

### Amyotrophic Lateral Sclerosis

Amyotrophic lateral sclerosis (ALS) is a neurodegenerative disease of motor neurons, motor cortex, and corticospinal tract linked with GSK-3β overactivity [28]. GSK3β activity shows an increase in various ALS models and patients. Furthermore, GSK3β inhibition can suppress the defective phenotypes in various ALS models [29]. GSK3β expression and cytosolic levels of GSK3β are augmented in the spinal cord and frontotemporal cortex of ALS patients [21]. Preclinical findings support that GSK-3β activity is increased in ALS animals and patients [29]. In addition, the expression of GSK-3β and catenin which reflect the activity of GSK-3β had been reported to be increased in the frontal cortex and hippocampus in ALS patients [30]. Activation of GSK-3β in ALS is related to the downregulation of PI3K, and PI3K activators may be effective in the management of ALS [31]. To identify the therapeutic potential of GSK3β-targeted drugs in ALS treatment, many studies have shown that GSK3β inhibitors can attenuate ALS disease progression. Valproic acid which is a mood stabilizer can indirectly inhibit GSK3β via Akt pathway. Valproic acid acts as a neuroprotective for motor neurons, delays disease progression, and extends life span in mouse ALS model [21]. A combination of lithium and valproic acid showed superior effects on motor dysfunction and disease progression in mouse ALS model by inhibiting GSK3β compared to lithium and valproic acid when used alone [32]. Therefore, GSK3β activity is increased in numerous ALS and GSK3β inhibition can rescue defective phenotypes of ALS in numerous models.

### Multiple Sclerosis

Multiple sclerosis (MS) is the most common demyelinating neurodegenerative disease of the central nervous system (CNS) in young adults [32, 33]. MS is regarded as an autoimmune disease causing injury of myelin sheath by immune cells and inhibiting the production of myelin. Oligodendrocytes which involved with the synthesis of the myelin sheath are typically affected in MS [33, 34]. In demyelinating diseases as in MS and experimental autoimmune encephalomyelitis (EAE) , GSK-3β activity is increased [20, 35]. It has been shown that the expression of GSK-3β is highly increased in MS patients mainly in the cerebral cortex and corpus callosum [15]. Higher expression of GSK-3β is increased in MS and induces the development of neuroinflammation by activating the release of pro-inflammatory cytokines via TLR4-dependent pathway [36, 37]. It has been established that GSK3β is intricate in Wnt-beta-catenin signaling, which participates to the inhibition of myelination and remyelination processes in humans [38]. dGSK3β rs334558 polymorphism is a susceptibility factor for MS, as it is found in the promoter region, a possible explanatory mechanism that could be an influence of the variant on the gene transcription rate [39]. Lithium treatment significantly delayed the onset of EAE and improves its severity by inhibiting pro-inflammatory TNF-α and inactivated GSK-3β [20]. Furthermore, lithium improves stem cell differentiation into oligodendrocytes and enhances re-myelination in MS [38]. Thus, exaggeration of GSK-3β is linked with MS neuropathology, and GSK-3β inhibitors may be effective in the management of MS.

Taken together, over-expression of GSK-3β is linked with the development of different neurodegenerative diseases, and GSK-3β inhibitors could be a novel therapeutic strategy in the management of neurodegenerative diseases.

## The Possible Role of GSK-3β in PD

It has been observed that GSK-3β over-activity is correlated with PD neuropathology by inducing neuroinflammation, derangement of blood-brain barrier (BBB) permeability, and degeneration of dopaminergic neurons in the SN [12]. A previous preclinical study found that 6-hydroxydopamine (6-OHDA)-induced dopaminergic degeneration is mediated by the expression of GSK-3β [40]. In 6-OHDA-induced PD, the activated GSK-3β not only induces degeneration of dopaminergic neurons but also blocks the proliferation and differentiation of neuron stem cells, thereby blocking neurogenesis [41]. Many studies have exposed that the inhibition of GSK-3β reduces dopaminergic neuron injury induced by MPTP toxicity, indicating the association of GSK-3β with the pathogenesis of PD [42]. Khan et al. [43] showed that GSK-3β accelerates neuroinflammation in PD by triggering the expression of pro-inflammatory cytokines. The harmful effect of GSK-3β activation on dopaminergic neuron survival was further established in transgenic mice expressing a constitutively active mutant of GSK-3β [40]. Dysregulation of GSK-3β results in aberrant mitochondrial function, which is implicated in PD [43]. Considerable evidence suggests that GSK-3β mediates glial cell activation and promotes the release of pro-inflammatory cytokines via regulating several transcriptional factors and development of neuroinflammation [44]. Moreover, GSK-3β is increased in the striatum of postmortem brains of PD patients [45]. GSK-3β was activated by phosphorylation at its Tyr216 in the striatum of PD patients [36]. Increased GSK-3β protein levels have also been reported in peripheral blood lymphocytes in PD patients [46]. Therefore, GSK-3β overactivity promotes PD neuropathology through induction of mitochondrial dysfunction and neuroinflammation.

Furthermore, GSK-3β may contribute to the formation of protein aggregates or intracellular inclusions in PD. Deficiency of autophagy-lysosomal pathway, leading to dysfunction of protein aggregate clearance, was observed in postmortem brains of PD patients [47]. GSK-3β may progressively lead to intracellular and axonal deposit in PD neuropathology [43, 47]. GSK-3β inhibits autophagy leading to reduction in the clearance of α-Syn .GSK-3β is known to be involved in neuronal development and suppression of GSK-3β showed the ability to reduce α-Syn in cellular models of PD [48]. It has been observed that exaggerated GSK-3β inhibits dopaminergic neurotransmission in the SN [12]. The dopamine D2 receptor regulates Akt and may also target the Wnt pathway, two signaling cascades that inhibit GSK-3β [12, 48]. In addition, abnormal dopaminergic activity is associated with PD due to overactivity of GSK‐3*β*. Inhibition of GSK‐3*β* has been reported to attenuate D1 receptor agonist‐induced hyperactivity in mice [49]. Synaptic loss is correlated with cognitive deficits in PD. Synaptic dysfunction leads to impairment of the balance between long-term potentiation (LTP) and long-term depression (LTD). Of note, LTP inhibits GSK-3β activity which required for LTD. Though the precise mechanism underlying this remains indistinct, it has been established that constitutive GSK-3β activity promotes basal AMPAR endocytosis leading to inhibition of synaptic plasticity and development of cognitive dysfunction in PD [43, 50]. Thus, exaggerated GSK‐3*β* can induce structural and functional alterations in the dopaminergic neurons of SN in PD.

As well, α-Syn activates the expression and forms a heterotrimeric complex with GSK-3β. The activation of GSK-3β was absolutely dependent on the presence of α-Syn, as indexed by the absence of p-GSK-3β in cells lacking α-Syn and in α-Syn knockout mice. In turn, GSK-3β promotes aggregation of α-Syn [51]. Autopsies from postmortem PD brains revealed that levels of phosphorylated GSK-3β were higher in PD patients as compared to healthy controls [52]. Evidence from preclinical and clinical studies revealed that GSK-3β expression is augmented in PD [53]. Of interest, GSK-3β polymorphism increases PD risk [54]. In a mouse model of tauopathy, GSK-3β expression is co-localized with α-Syn in the striatum [55]. Remarkably, a neuroprotective protein progranulin which is highly reduced in PD modulates the expression of GSK-3β [56, 57]. Mutation of progranulin is associated with over-expression of GSK-3β and the development of PD [57].

The activation of Nrf2 enhances the expression of ARE and hemeoxygenase-1 (HO-1), which decreases excessive cellular stress, mitochondrial dysfunction, apoptosis, and neuronal degeneration, which is the major cause of motor dysfunction including PD [58]. Therefore, there is a link between GSK-3β and the Nrf2/HO-1 signaling pathway in PD [58]. Over-expression of the GSK-3β and downregulation of the Nrf2/ARE pathway are responsible for a decrease in anti-oxidant defense effects. These underline the usefulness of dual GSK-3β inhibitors/Nrf2 inducers. Thus, a dual modulator, the structures of a curcumin-based analogue, as GSK-3β inhibitor, and a diethyl fumarate fragment, as Nrf2 inducer, could be effective in PD [59]. These preclinical and clinical findings proposed that over-expression of GSK-3β are linked with the pathogenesis of PD. Though, the mechanisms by which GSK-3β promotes PD neuropathology are not well elucidated.

## Mechanistic Role of GSK-3β in PD

### Mitochondrial Dysfunction and Oxidative Stress

Reactive oxygen species (ROS) are produced continuously by all body tissues that are eliminated by endogenous anti-oxidant capacity [60–62]. When there is an imbalance between ROS generation and anti-oxidant capacity, oxidative stress is developed [63]. The mitochondria are the major site for the generation of ROS which affect mitochondrial DNA leading to more ROS generation. It has been reported that oxidative stress plays a critical role in the degeneration of dopaminergic neurons in the SN [64]. Of note, dopamine turnover and environmental neurotoxins induce mitochondrial dysfunction which promotes the development and progression of oxidative stress [65]. Dopamine outside the synaptic vesicle undergoes auto-oxidation or is metabolized by monoamine oxidase (MAO) to form ROS which induces mitochondrial dysfunction [66]. Interestingly, mitochondrial dysfunction is highly related to augments ROS generation in PD [67]. Importantly, complex I deficiency of the respiratory chain is associated with the pathogenesis of PD [64]. In the experimental PD model, rotenone or MPTP can induce inhibition of mitochondrial complex I and reduce ATP formation with subsequent injury and degeneration of dopaminergic neurons in the SN [68]. In clinical settings, oxidative stress biomarkers were reported to be increased in PD patients compared to healthy controls [69]. Overall, these findings indicated that mitochondrial dysfunction and oxidative stress are closely related to PD neuropathology.

**Fig. 3:**
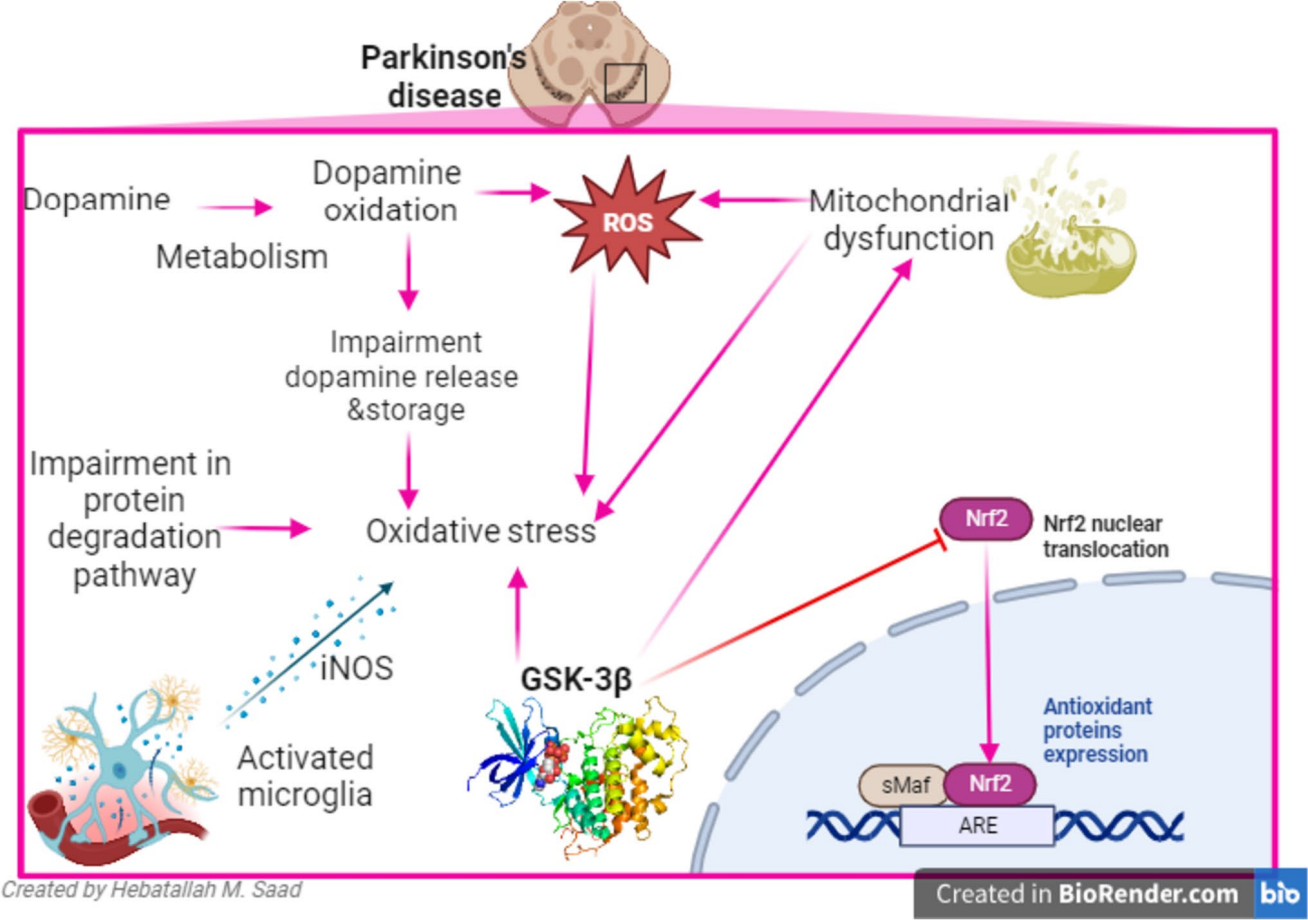
Role of GSK-3β in PD: oxidative stress is a central mechanism intricate in the pathogenesis of PD. Dopamine metabolism and mitochondrial dysfunction induce formation of reactive oxygen species (ROS) which causes oxidative stress. Beside, impairment of protein degradation pathway promotes oxidative stress. Activated microglia and GSK-3β accelerate oxidative stress by inhibiting the expression of anti-oxidant proteins such as nuclear factor-related erythroid factor (Nrf2) with inhibition of anti-oxidant response element (ARE)

On the other hand, GSK-3β is intricate in the pathogenesis of PD through modulation of mitochondrial dysfunction and oxidative stress [70]. In vitro study demonstrated that oxidative stress promotes the expression of GSK-3β which inhibits transcription of anti-oxidant Nrf2 leading to propagation of oxidative stress-induced neuronal injury [70]. Liu et al. [71] observed that inhibition of GSK-3β attenuates oxidative stress-induced kidney injury in rats by upregulation of the Nrf2 signaling pathway. Likewise, insulin and IGF-1 inhibit oxidative stress in rat cortical neurons by reducing the expression of GSK-3β [72]. In vivo and in vitro studies confirmed that inhibition of GSK-3β attenuates the development and progression of mitochondrial dysfunction and oxidative stress in mice with muscle dysfunction [73]. These verdicts proposed that GSK-3β over-activity induces the development of PD by triggering mitochondrial dysfunction and oxidative stress in the dopaminergic neurons of the SN (Fig. 3).

### Inflammatory Signaling Pathways

It has been reported that different inflammatory signaling such as nuclear factor kappa B (NF-κB) and nod-like receptor pyrin 3 (NLRP3) inflammasome are intricate in the pathogenesis of PD [4]. NF-κB is an inflammatory signaling protein that promotes the expression and release of chemokines and pro-inflammatory cytokines. NF-κB is involved in the regulation of cell differentiation, proliferation, apoptosis, and innate and adaptive immune response [74]. It has been reported that NF-κB can induce degeneration of dopaminergic neurons in the SN [75]. Aging-induced immune dysregulation promotes NF-κB expression and associated degeneration of dopaminergic neurons in the SN [75].

Notoriously, released α-Syn from injured neurons triggers NF-κB activation and expression of pro-inflammatory cytokines. In injured neuron triggers NF-κB activation, and induces further degeneration of dopaminergic neurons in the SN [75]. Besides, exaggerated GSK-3β signaling activates the expression of NF-κB as confirmed in an in vitro study [76]. In an experimental study, inhibition of GSK-3β by selective inhibitors decrease NF-κB activation in rats [77].

Furthermore, NLRP3 inflammasome is a multiprotein complex involved in the release of IL-1β and IL-18 via caspase activation [78]. NLRP3 inflammasome is activated by various stimuli in conical and non-conical pathways. NLRP3 inflammasome is regarded as a metabolic sensor detects inflammatory and oxidative stress injury [78]. Different studies revealed that activated NLRP3 inflammasome signaling pathway triggers the release of pro-inflammatory cytokines, development of neuroinflammation, and degeneration of dopaminergic neurons in the SN [79, 80]. Furthermore, NLRP3 inflammasome-induced pyroptosis could be the potential mechanism for the development of PD. Indeed, NLRP3 inflammasome interacts with α-Syn leading to progressive neuronal degeneration. Therefore, NLRP3 inflammasome level is correlated with α-Syn level in PD patients [81]. It has been shown that GSK-3β triggers the expression of NLRP3 inflammasome leading to pyroptosis [82]. Besides, GSK-3β via inhibition of the Nrf2 signaling pathway promotes oxidative stress which enhances activation of NLRP3 inflammasome [83].

**Fig. 4:**
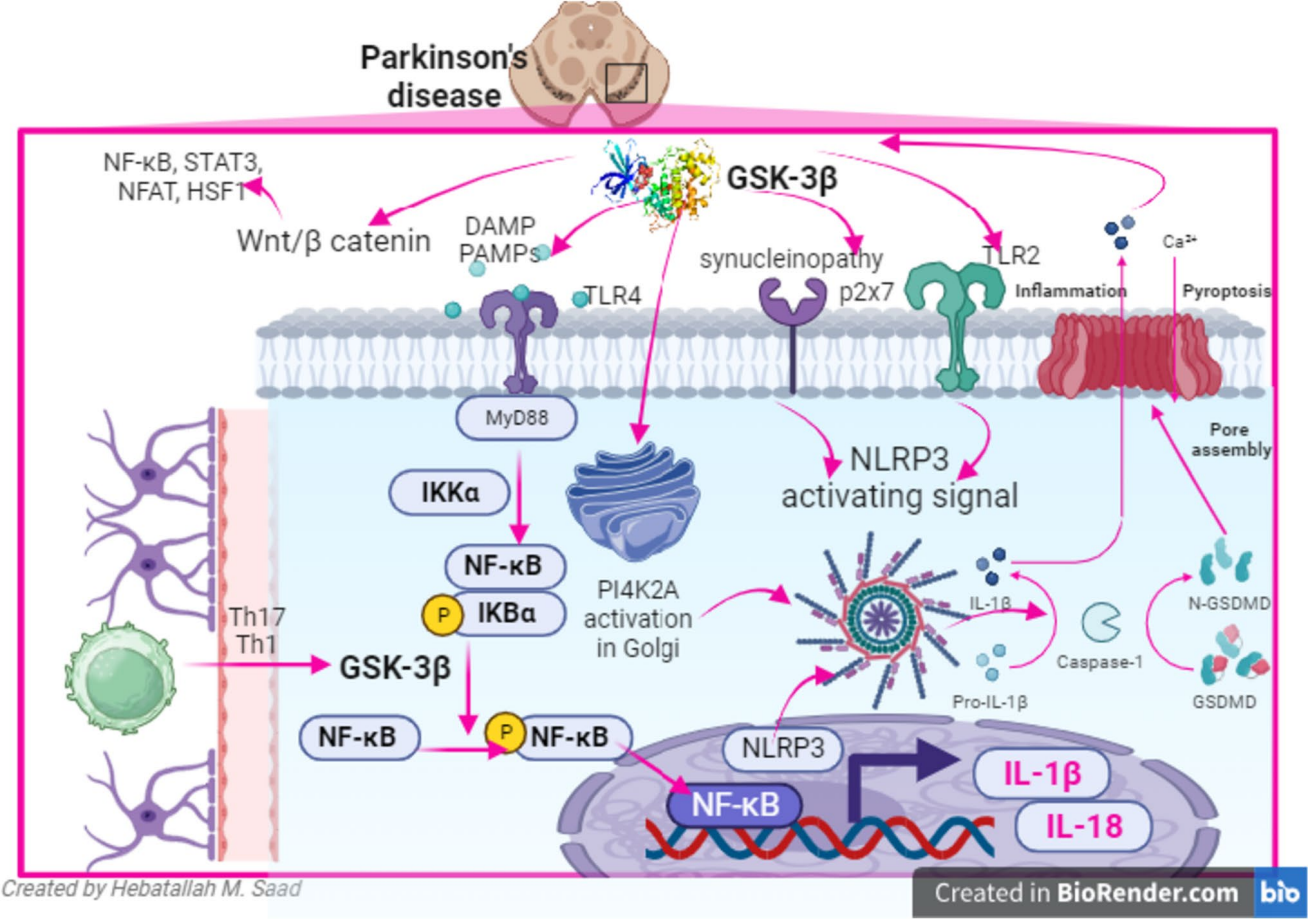
Inflammatory signaling pathways and GSK-3β in PD: GSK-3β triggers the interaction between pathogen‐associated molecular pattern molecules (PAMP) and damage-associated molecular pattern (DAMP) with toll-like receptor 4 (TLR4) which promote NF-κB which induce the expression of NLRP3 inflammasome leading to the release of pro-inflammatory cytokines. GSK-3β via activation of TLR2 and p2 × 7 also induce the expression of NLRP3 inflammasome

Therefore, NF-κB and NLRP3 inflammasome are activated in response to over-activated GSK-3β in PD leading to progressive neuronal injury (Fig. 4).

### Neuroinflammation

Neuroinflammation is an immune response of the CNS to exogenous infectious agents or endogenous stress stimuli as in many neurological disorders such as neurodegenerative diseases [84]. Microglia and astrocytes are intricate in the development of neuroinflammation; nevertheless, peripheral immune cells which traverse injured BBB can involve the development of neuroinflammation in chronic inflammatory disorders [85]. Neuroinflammation in the acute phase is defended to eradicate the underlying cause; however, chronic neuroinflammation may induce neuronal injury, synaptic dysfunction, and exacerbation of brain neuropathology [86]. Different genetic and epidemiological studies confirmed the potential role of neuroinflammation in PD neuropathology [48]. Postmortem study revealed that microglia and T cells are highly concentrated in the SN of PD brains due to dysregulation of innate and adaptive immune responses [87]. Evidence from preclinical studies showed that neuroinflammation is correlated with progressive degeneration of dopaminergic neurons in the SN [88]. Findings from postmortem analysis illustrated that levels of pro-inflammatory cytokines in the CSF were increased in PD patients compared to healthy controls [88]. Pro-inflammatory cytokines activate inflammatory signaling pathways leading to oxidative stress injury of dopaminergic neurons in the SN [88]. Remarkably, Th1 and Th17 enhance MPTP-mediated injury of dopaminergic neurons in the SN [89]. As well, neuroinflammatory biomarkers are increased in PD patients compared to healthy controls [90]. Besides, many studies highlighted that GSK-3β promotes the progression of neuroinflammation by inducing the expression of inflammatory signaling pathways and pro-inflammatory cytokines [43, 91]. Furthermore, different preclinical studies confirmed that inhibition of GSK-3β leads to attenuation of neuroinflammation in different neurodegenerative disorders including PD [92, 93]. For example, Lee et al. [94] revealed that inhibition of GSK-3β by specific peptide attenuates nigrostriatal neurodegeneration in rat PD models. These findings proposed that GSK-3β plays a crucial role in the development and progression of neuroinflammation in PD.

### Brain-Derived Neurotrophic Factor

Brain-derived neurotrophic factor (BDNF) is a member of the neurotrophin protein family concerned in the resistance toward neuronal injury [95]. BDNF acts on tyrosine kinase receptor B (TrkB) and p75NT receptor (p75NTR) [92]. BDNF is released from specific brain regions including the hypothalamus, hippocampus, and limbic system [96–98]. It has been shown that BDNF serum level is reduced in PD patients compared to healthy controls [99]. However, in advanced stages of PD neuropathology BDNF serum level is increased as a compensatory mechanism to mitigate oxidative and inflammatory disorders [99]. Chang et al. [100] confirmed that activation of BDNF signaling reduces motor deficit and cognitive dysfunction in the mouse PD model. Improvement of BDNF signaling by anti-depressants promotes cognitive and motor functions in PD patients [101].

In relation to GSK-3β, BDNF inhibits GSK-3β activity by increasing PI3K in neural stem cells [102]. Likewise, BDNF attenuates phencyclidine-induced apoptosis via activation of PI3K and inhibition of GSK-3β in cultured cortico-striatal neurons [103]. Of note, GSK-3β-induced neuropsychiatric disorders are mediated by inhibition of BDNF signaling [104]. These findings proposed a reciprocal relationship between BDNF and GSK-3β. Therefore, higher expression of GSK-3β in the early stages of PD neuropathology might contribute in the reduction of BDNF leading to progressive neuronal injury.

## GSK-3β Inhibitors in PD

GSK-3β is a central point in a number of signaling pathways in the pathogenesis of this neurodegenerative disease, affecting multiple pathological events involved in dopaminergic neuron degeneration, thus providing a potential target in the therapeutic management by blocking the pathogenic pathways involved in PD pathogenesis. GSK-3 inhibition has been considered a potential therapeutic strategy for PD treatment [12]. In the last decades the scientific community has been working to understand the role of GSK-3 with the aim in mind of design efficient and selectivity GSK-3 inhibitors. However, so far clinical and preclinical GSK-3 inhibitors have been both sub-optimal regarding potency, poor GSK-3β selectivity over other CNS targets and closely related kinases, low CNS exposure, and chronic toxicity. Research into GSK-3β inhibitors relay primarily on identification of the new use of the known GSK-3β inhibitors and the development of them in order to improve selectivity and toxicity. Many efforts have been done for using of GSK-3β inhibitors in the management of neurodegenerative diseases including PD [105]. GSK-3β inhibitors are categorized into 4 classes according to Ruiz et al. [106]: (I) cationic GSK-3β inhibitors including lithium, copper, and zinc; (II) ATP blockers which inhibit ATP binding kinase such as synthetic organic molecules; (III) allosteric inhibitors; and (IV) substrate competitive inhibitors. However, synthetic GSK-3β inhibitors are non-specific that may inhibit other kinases. In addition, drug resistance is higher among GSK-3β inhibitors due to the mutation of ATP binding sites of GSK-3β [107]. Most of the clinical trials for the use and safety of GSK-3β inhibitors were conflicting and did not reach Phase III [108]. Since GSK-3β has a critical role in metabolism, insulin signaling, protein regulation, and inflammation, GSK-3β inhibition is regarded as an attractive target for therapeutic intervention in metabolic and neurodegenerative diseases [105]. Though, the design of specific inhibitors of intracellular kinases including GSK-3β is very difficult because the kinase families share conserved ATP-binding sites, and consequently, currently developed kinase inhibitors have mostly off-target effects [109]. Since GSK-3β is a constitutively active kinase, extreme GSK-3β inhibition could have adverse effects by disrupting its physiological roles. For example, SB216763, a well-known GSK-3β inhibitor, was effective in attenuating Aβ-induced neurotoxicity in an AD model, but it induced neuronal death, gliosis, and behavioral deficits in control animals [110]. Thus, it would be important to design a GSK-3β inhibitor to selectively inhibit the activity of the kinase when it is excessively activated in a pathological condition without affecting its physiological roles in normal condition.

Additional significant challenge to overcome for a GSK-3β inhibitors to be converted in an effective drug for PD treatment is its specific brain distribution. The drug needs to cross the BBB to exert its action in the regulation of exacerbated GSK-3β brain levels. Usually this is not an easy task for GSK-3β inhibitors when oral bioavailability is the preferred administration route for chronic PD treatment. It is very difficult to balance the equilibrium between molecular lipophilicity to enter into the brain and molecular hydrophilicity to be orally administrated. That reason has ruled out several promising GSK-3β inhibitors into the market. Determination of potential brain penetration should be incorporated in the first stages of GSK-3β inhibitor development. Therefore, GSK-3β inhibitors which cannot cross BBB have limited efficacy in the management of neurodegenerative diseases [111]. Despite these findings, different GSK-3β inhibitors reached the market for the treatment of different diseases.

Interestingly, clinical side effects of GSK-3β inhibitors are rather scarce since a limited number of GSK-3β inhibitors have reached the clinical phase [112]. GSK-3β may lead to hyperglycemia by inhibiting the conversion of glucose to glycogen via inhibition of glycogen synthase [113]. This function is modulated by insulin which activates glycogen synthase and inhibits GSK-3β activity by about 50% [72]. Furthermore, GSK-3β inhibitors are of distinct chemical structures and thus differ in their bio-clinical and pharmacological properties. Thus, it is difficult to decide at this point what adverse events will be commonly associated with inhibition of GSK-3β inhibitors [112]. Lithium is the only GSK-3β inhibitor that has been in clinical use for a significant time. Though, lithium lacks target specificity, and its adverse side effects and high toxicity do not necessarily reflect events linked with inhibition of GSK-3β [114]. AZD-1080 and NP-12/Tideglusib (Noscria) reached the clinic in 2006. AZD-1080 was withdrawn due to nephrotoxicity observed in phase I clinical trials [115]. NP-12 in phase IIb trials for AD and paralysis supranuclear palsy and no side effects/off targets effects have been described at this time [116]. Their discrete chemical structures and/or different inhibition mode are most likely responsible for the different clinical impacts observed with these two compounds [115, 116]. Results from TAURUS and ARGO studies will disclose the safety and efficacy of tideglusib in humans [117]. In the meantime, an increasing number of GSK-3β inhibitors are being tested in preclinical models, and it is anticipated that some will enter clinical trials [118].

Of note, concerns had been raised regarding the potential toxicity of GSK-3β inhibitors ranging from hypoglycemia to tumorigenesis and neuron deregulation [119]. GSK-3β is vital for life, and there is a disquiet that its inhibition could prevent cells from functioning normally. However, GSK-3β activity is elevated in pathological conditions; thus, a smooth inhibition of GSK-3 able to restore down levels of activity to physiological ones would be enough to produce an important therapeutic effects in diseases, being that point crucial for not producing adverse effects. Therefore, GSK-3β inhibitors increase insulin sensitivity and may increase cell proliferation via Wnt signaling-dependent pathway [119]. In addition, the interaction of Wnt/β-catenin due to GSK-3β inhibition promotes oncogene transcription and increases the risk of malignancy [120]. Activation of the proto-oncogenic molecule β-catenin by inhibition of GSK-3 is another major concern claiming that long-term inhibition of GSK-3β may promote cancer. However, no direct in vivo evidence has indicated tumorigenesis upon administration of GSK-3β inhibitors. On the contrary, in certain cancers GSK-3β inhibitors reduced cell proliferation and enhanced cell death upon irradiation treatment [120].

However, GSK-3β inhibitor lithium which was used for a long time in the management of bipolar disorders was not associated with hypoglycemia and malignancies [121]. Lithium inhibits 25% of GSK-3β without effect on Wnt/β-catenin signaling which increases cell proliferation [121].

Therefore, repurposing of other drugs with well-known pharmacokinetic/pharmacodynamic profiles that have inhibitory effects on GSK-3β seems to be more appropriate in the management of PD.

### Lithium

Lithium is a chemical element present as pegmatic mineral. Lithium salts are widely used in the management of various neurological disorders including mania, bipolar disorders, AD, and schizophrenia [122]. It was approved by FDA in 1970 for use in the management of bipolar disorders. Lithium was first used in the nineteenth century for the treatment of gout [123]. In 1949, lithium was re-introduced in treating mania and other bipolar disorders [123]. The main mechanism of action of lithium is related to increasing serotonin synthesis and inhibits the synthesis of norepinephrine [124]. The fundamental mechanism of lithium is through inhibition of inositol monophosphatase which is required for conversion of inositol monophosphate to inositol which is implicated in the pathogenesis of bipolar disorders [124]. As well, lithium blocks GSK-3β directly or indirectly via inhibition of the mechanistic target of rapamycin (mTOR) which is a necessary downstream signaling of GSK-3β [121]. In mania, GSK-3β activity is augmented by the over-activity of dopamine signaling, leading to inhibition of both cAMP-response element binding protein (CREB) and β-catenin. Furthermore, lithium inhibits both NO signaling and NMDA receptors [125]. Different preclinical studies revealed that lithium can inhibit GSK-3β and prevents the accumulation of tau protein in AD mouse model [126]. As well, lithium attenuates MPTP-induced dopaminergic neuronal injury in PD mouse model [127]. The underlying neuroprotective effect of lithium, in PD, is related to the inhibition of GSK-3β and oxidative and activation of neuroprotective BDNF [127]. In virtue of its anti-oxidant and neuroprotective effects, lithium seems to be effective in PD. However, due to its relative toxicity and wide-spectrum adverse effects, a large dose of lithium is not appropriate monotherapy in the management of PD. Thus, low non-toxic dose of lithium in combination with other anti-PD agents seems more effective. Of note, only one ongoing trial using lithium in PD illustrated that medium-dose lithium improves disease progression measured by brain MRI, but it is poorly tolerated by 33% of PD patients [128]. Further PD clinical research is merited examining lithium’s tolerability; effects on biomarkers and potential disease-modifying effects are recommended.

### Famotidine

Famotidine is an H2 blocker used in the management of peptic ulcers and gastroesophageal reflux disease. It was patented in 1979 and become available in the market in 1985. Famotidine is a rapid-acting drug with minimal adverse effects, though a large therapeutic dose of it may cause seizures [129, 130]. It has been shown that famotidine has a neuroprotective effect by inhibiting GSK-3β expression in MK-801-induced toxicity in SH-SY5Y cell line [131]. In addition, famotidine attenuates ketamine-induced schizophrenic behavior in rats by inhibiting GSK-3β [132]. A previous pilot study on 7 PD patients revealed that daily intake of 80 mg/day of famotidine for 6 weeks improve motor [133]. Of interest, famotidine enhances the therapeutic efficacy of levodopa and improves non-motor symptoms in PD patients [134]. However, famotidine has no clinical benefit against the development of levodopa-induced dyskinesia [135].

### Naproxen

Naproxen is an analgesic and anti-inflammatory drug belonging to the non-steroidal anti-inflammatory drug (NSAID). It acts by reversible inhibition of cyclooxygenase enzymes (COXs), i.e., non-selective COX inhibitors [136]. It has been reported by preclinical investigations that naproxen has anti-diabetic effects by inhibiting GSK-3β activity [137]. Furthermore, naproxen attenuates carcinogenesis by inhibiting GSK-3β and modulation of Wnt/β-catenin signaling [137]. As well, naproxen has an anti-cancer effect via inhibition of GSK-3β [138]. In general, NSAIDs have neuroprotective effects against PD neuropathology by inhibiting neuroinflammation and abnormal immune response. In addition, non-selective COX inhibitor ibuprofen also has a chemo-preventive efficacy against cancer by inhibiting GSK-3β and modulating Wnt/β-catenin signaling [139]. Notably, ibuprofen was reported to be effective in the management of PD [140]. Therefore, NSAIDs with inhibitory effects on GSK-3β could be effective in the management of PD.

### Metformin

Metformin is an insulin-sensitizing drug used as first-line therapy in the management of type 2 diabetes (T2D) [141]. Metformin has pleiotropic effects through modulation of inflammation and oxidative stress [141]. A recent study conducted by Alrouji et al. [142] suggested that metformin has a double-sword effect against PD neuropathology. The neuroprotective effect of metformin against PD is through inhibition of inflammation and oxidative stress. However, its detrimental effect is related to the development of B12 deficiency and hyperhomocysteinemia [142]. Nevertheless, prolonged use of metformin seems to be protective rather than harmful [143]. On the other hand, metformin has a cytoprotective effect by inhibiting the expression and activity of GSK-3β in non-small-cell lung cancer [144]. Similarly, metformin attenuates mitochondrial dysfunction and associated oxidative stress by inhibiting GSK-3β in preosteoblast [145]. Metformin acts by activating AMPK [146] and AMPK activators have been observed to protect dopaminergic neurons in the SN [147]. It has been shown that AMPK activator GSK621 attenuates MPTP mouse PD model. AMPK activator GSK621 dramatically ameliorated PD by increasing the levels of dopamine and rescuing the loss of dopaminergic neurons, which is dependent on the mitochondrial pathway. Regulation of AMPK/GSK-3β/PP2A pathway-related proteins by GSK621 was partially inhibited the development of PD, suggesting that a negative feedback loop exists between AMPK action and mitochondrial dysfunction-mediated apoptosis. Therefore, mitochondrial dysfunction and apoptosis in the pathogenesis of PD might be mediated by AMPK/GSK-3β/PP2A pathway action, which might be a promising new option for future therapy of PD [147]. Moreover, flavonoid dihydromyricetin has a potent anti-oxidative agent against MPTP-induced behavioral impairment in mice by inhibiting GSK-3β through AMPK-dependent pathway [148].

Therefore, the neuroprotective effect of metformin against PD may be mediated by inhibiting GSK-3β.

### Tideglusib

Tideglusib is a small molecule that inhibits GSK-3β irreversibly. It is regarded as a non-ATP competitive inhibitor of GSK-3β used in different neurodegenerative diseases [107]. Tideglusib has a neuroprotective effect against MPTP-induced dopaminergic injury in mice in a dose-dependent manner via inhibition of GSK-3β [46]. Likewise, tideglusib attenuates 6-OHDA and lipopolysaccharide (LPS) PD animal model by inhibiting GSK-3β in the dopaminergic neurons of the SN [149]. Furthermore, tideglusib reduces oxidative stress in the dopaminergic neurons of the SN by inducing the expression of anti-oxidant enzymes [150]. In addition, tideglusib can decrease the risk of progressive dopaminergic neurodegeneration induced by neuroinflammation which is augmented in response to GSK-3β [151]. Therefore, tideglusib seems to be effective in PD. In addition, tideglusib has also shown acceptable safety and was well tolerated in several chronic clinical trials regarding different neurological diseases [152–157]. However, tideglusib failed in a Phase II clinical trial of AD due to no clinical benefits in cognitive improvement despite its neuroprotection in preclinical AD models [155]. Therefore, intervention at an earlier disease stage, longer duration of treatment, and better dosing of tideglusib should be taken into account for future clinical trials that should also be considered in clinical trials for PD, which may possibly be confronted with similar problems.

Taken together, GSK-3β inhibitors could be effective in PD by reducing inflammatory and oxidative stress disorders which are associated with degeneration of dopaminergic neurodegeneration.

## Future Research and Perspective

Most of the recently developed GSK-3β inhibitors fall into the ATP competitive inhibitors which are characterized by good safety and low specificity but tend to induce drug resistance. Although their discovery is more challenging, compounds that recognize other regions of the kinase are considered a favorable choice as the target is more conserved. Therefore, GSK-3β inhibitors mainly lithium and tideglusib could be effective as adjuvant treatments in the management of PD by reducing neuroinflammation and degeneration of dopaminergic neurons in the SN [106]. A combination of lithium plus L-DOPA could be a substantial combination in the management of PD. It has been reported that lithium in combination with L-DOPA play not only as a neuroprotectant, but also for reducing abnormal involuntary movements and possibly alleviating potential side effects associated with the current treatment for PD [158]. However, chronic lithium use is associated with an increased incidence of dopaminergic drug use compared with anti-depressants, identifying a prescribing cascade related to lithium use in the elderly. Whether this reflects inappropriate treatment of action tremor or treatment of drug-induced Parkinsonism should be evaluated by a close examination of prescribing practices [159]. Moreover, lithium has hypoglycemic effect and improves the function of pancreatic β cells through inhibition of GSK-3β [160]. Likewise, anti-diabetic metformin which has an inhibitory effect on GSK-3β [147] may reduce PD neuropathology. Thus, GSK-3β inhibitors could be more effective in PD with associated comorbidities such T2D and psychiatric disorders.

Therefore, selective use of GSK-3β inhibitors with good efficacy and high safety in combination with anti-PD medications might be a novel therapeutic strategy in the management of PD.

## Conclusions

PD is a progressive neurodegenerative disease of the brain may be linked with over-activation of GSK-3β which is a conserved threonine/serine kinase protein involved in the regulation of cellular anabolic and catabolic pathways. Over-expression of GSK-3β is also linked with the development of neurodegenerative diseases such as AD, ALS, and MS. NF-κB and NLRP3 inflammasome are activated in response to dysregulated GSK-3β in PD leading to progressive neuronal injury. Higher expression of GSK-3β in the early stages of PD neuropathology might contribute to the reduction of neuroprotective BDNF. Thus, GSK-3β inhibitors could be effective in PD by reducing inflammatory and oxidative stress disorders which are associated with dopaminergic neurodegeneration. Furthermore, preclinical and large-scale prospective studies are warranted in this regard.

## Data Availability

Data sharing not applicable to this article as no data sets were generated or analyzed during the current study.

## References

[1] Al-kuraishy HM, Al-Gareeb AI, Kaushik A, Kujawska M, Ahmed EA, Batiha GES (2023) SARS-COV-2 infection and Parkinson’s disease: possible links and perspectives. J Neurosci Res 101(6):952–975 36717481 10.1002/jnr.25171

[2] Al-Kuraishy HM, Jabir MS, Al-Gareeb AI, Albuhadily AK (2023) The conceivable role of prolactin hormone in Parkinson disease: the same goal but with different ways. Ageing Res Rev 91:102075. 10.1016/j.arr.2023.102075 37714384 10.1016/j.arr.2023.102075

[3] Heras-Garvin A, Stefanova N (2020) From synaptic protein to prion: the long and controversial journey of α-synuclein. Front Synaptic Neurosci 12:584536 33071772 10.3389/fnsyn.2020.584536 PMC7536368

[4] Alrouji M, Al-kuraishy HM, Al-Gareeb AI, Alexiou A, Papadakis M, Jabir MS et al (2023) NF-κB/NLRP3 inflammasome axis and risk of Parkinson’s disease in type 2 diabetes mellitus: a narrative review and new perspective. J Cell Mol Med 27(13):1775–1789. 10.1111/jcmm.17784 37210624 10.1111/jcmm.17784 PMC10315781

[5] Xu B, Fan F, Liu Y, Liu Y, Zhou L, Yu H (2023) Distinct effects of familial Parkinson’s disease-associated mutations on α-synuclein phase separation and amyloid aggregation. Biomolecules 13(5):726 37238596 10.3390/biom13050726 PMC10216457

[6] Airavaara M, Parkkinen I, Konovalova J, Albert K, Chmielarz P, Domanskyi A (2020) Back and to the future: from neurotoxin-induced to human Parkinson’s disease models. Curr Protoc Neurosci 91(1):e88 32049438 10.1002/cpns.88

[7] Chung SJ, Yoo HS, Lee YH, Lee PH, Sohn YH (2019) Heterogeneous patterns of striatal dopamine loss in patients with young-versus old-onset Parkinson’s disease: impact on clinical features. J Mov Disord 12(2):113 31158944 10.14802/jmd.18064 PMC6547040

[8] Consonni A, Miglietti M, De Luca CMG, Cazzaniga FA, Ciullini A, Dellarole IL et al (2022) Approaching the gut and nasal microbiota in Parkinson’s disease in the era of the seed amplification assays. Brain Sci 12(11):1579 36421902 10.3390/brainsci12111579 PMC9688507

[9] Hou X, Watzlawik JO, Fiesel FC, Springer W (2020) Autophagy in Parkinson’s disease. J Mol Biol 432(8):2651–2672 32061929 10.1016/j.jmb.2020.01.037 PMC7211126

[10] Li K-L, Huang H-Y, Ren H, Yang X-L (2022) Role of exosomes in the pathogenesis of inflammation in Parkinson’s disease. Neural Regen Res 17(9):1898–1906. 10.4103/1673-5374.335143 35142665 10.4103/1673-5374.335143 PMC8848593

[11] Kung H-C, Lin K-J, Kung C-T, Lin T-K (2021) Oxidative stress, mitochondrial dysfunction, and neuroprotection of polyphenols with respect to resveratrol in Parkinson’s disease. Biomedicines 9(8):918 34440122 10.3390/biomedicines9080918 PMC8389563

[12] Golpich M, Amini E, Hemmati F, Ibrahim NM, Rahmani B, Mohamed Z et al (2015) Glycogen synthase kinase-3 beta (GSK-3β) signaling: implications for Parkinson’s disease. Pharmacol Res 97:16–26 25829335 10.1016/j.phrs.2015.03.010

[13] Kaidanovich-Beilin O, Woodgett JR (2011) GSK-3: functional insights from cell biology and animal models. Front Mol Neurosci 4:40 22110425 10.3389/fnmol.2011.00040 PMC3217193

[14] Manduca JD, Thériault R-K, Perreault ML (2020) Glycogen synthase kinase-3: the missing link to aberrant circuit function in disorders of cognitive dysfunction? Pharmacol Res 157:104819 32305493 10.1016/j.phrs.2020.104819

[15] Noori T, Dehpour AR, Sureda A, Fakhri S, Sobarzo-Sanchez E, Farzaei MH et al (2020) The role of glycogen synthase kinase 3 beta in multiple sclerosis. Biomed Pharmacother 132:110874 33080467 10.1016/j.biopha.2020.110874

[16] Lee YH, Choi H-J, Kim JY, Kim J-E, Lee J-H, Cho S-H et al (2021) Ginsenoside Rg4 enhances the inductive effects of human dermal papilla spheres on hair growth via the AKT/GSK-3β/β-catenin signaling pathway. J Microbiol Biotechnol 31(7):933 34099599 10.4014/jmb.2101.01032 PMC9706015

[17] Jianing L, Shiqun S, Jia L, Xuetao Z, Zehua L, Tong S et al (2021) NCAPG, mediated by miR-378a-3p, regulates cell proliferation, cell cycle progression, and apoptosis of oral squamous cell carcinoma through the GSK-3β/β-catenin signaling. Neoplasma 68(6):1201–1211. 10.4149/neo_2021_210421N544 34585587 10.4149/neo_2021_210421N544

[18] Feng J, Xie L, Yu X, Liu C, Dong H, Lu W et al (2021) Perilipin 5 ameliorates high-glucose-induced podocyte injury via Akt/GSK-3β/Nrf2-mediated suppression of apoptosis, oxidative stress, and inflammation. Biochem Biophys Res Commun 544:22–30 33516878 10.1016/j.bbrc.2021.01.069

[19] Llorens-Marítin M, Jurado J, Hernández F, Ávila J (2014) GSK-3β, a pivotal kinase in Alzheimer disease. Front Mol Neurosci 7:46 24904272 10.3389/fnmol.2014.00046 PMC4033045

[20] Ahn M, Kim J, Park C, Cho J, Jee Y, Jung K et al (2017) Potential involvement of glycogen synthase kinase (GSK)-3β in a rat model of multiple sclerosis: evidenced by lithium treatment. Anat Cell Biol 50(1):48–59 28417055 10.5115/acb.2017.50.1.48 PMC5386926

[21] Ting H-C, Yang H-I, Harn H-J, Chiu I-M, Su H-L, Li X et al (2021) Coactivation of GSK3β and IGF-1 attenuates amyotrophic lateral sclerosis nerve fiber cytopathies in SOD1 mutant patient-derived motor neurons. Cells 10(10):2773 34685754 10.3390/cells10102773 PMC8535155

[22] Alsubaie N, Al-Kuraishy HM, Al-Gareeb AI, Alharbi B, De Waard M, Sabatier J-M et al (2022) Statins use in Alzheimer disease: bane or boon from frantic search and narrative review. Brain Sci 12(10):1290 36291224 10.3390/brainsci12101290 PMC9599431

[23] Al-Kuraishy HM, Al-Gareeb AI, Saad HM, Batiha GE-S (2023) Benzodiazepines in Alzheimer’s disease: beneficial or detrimental effects. Inflammopharmacology 31(1):221–230 36418599 10.1007/s10787-022-01099-4

[24] Leroy K, Yilmaz Z, Brion JP (2007) Increased level of active GSK-3β in Alzheimer’s disease and accumulation in argyrophilic grains and in neurones at different stages of neurofibrillary degeneration. Neuropathol Appl Neurobiol 33(1):43–55 17239007 10.1111/j.1365-2990.2006.00795.x

[25] Zhang Y, Huang N-Q, Yan F, Jin H, Zhou S-Y, Shi J-S et al (2018) Diabetes mellitus and Alzheimer’s disease: GSK-3β as a potential link. Behav Brain Res 339:57–65 29158110 10.1016/j.bbr.2017.11.015

[26] Yang Y, Wang L, Zhang C, Guo Y, Li J, Wu C et al (2022) Ginsenoside Rg1 improves Alzheimer’s disease by regulating oxidative stress, apoptosis, and neuroinflammation through Wnt/GSK-3β/β-catenin signaling pathway. Chem Biol Drug Des 99(6):884–896 35313087 10.1111/cbdd.14041

[27] Zhang H, Wang X, Xu P, Ji X, Chi T, Liu P et al (2020) Tolfenamic acid inhibits GSK-3β and PP2A mediated tau hyperphosphorylation in Alzheimer’s disease models. J Physiol Sci 70:1–11 32517647 10.1186/s12576-020-00757-y PMC10717460

[28] Koh S-H, Baek W, Kim SH (2011) Brief review of the role of glycogen synthase kinase-3β in amyotrophic lateral sclerosis. Neurol Res Int 2011:205761. 10.1155/2011/205761 21603026 10.1155/2011/205761 PMC3096311

[29] Hu JH, Zhang H, Wagey R, Krieger C, Pelech S (2003) Protein kinase and protein phosphatase expression in amyotrophic lateral sclerosis spinal cord. J Neurochem 85(2):432–442 12675919 10.1046/j.1471-4159.2003.01670.x

[30] Yang W, Leystra-Lantz C, Strong MJ (2008) Upregulation of GSK3β expression in frontal and temporal cortex in ALS with cognitive impairment (ALSci). Brain Res 1196:131–139 18221734 10.1016/j.brainres.2007.12.031

[31] Tolosa L, Mir M, Olmos G, Llado J (2009) Vascular endothelial growth factor protects motoneurons from serum deprivation-induced cell death through phosphatidylinositol 3-kinase-mediated p38 mitogen-activated protein kinase inhibition. Neuroscience 158(4):1348–1355 19041930 10.1016/j.neuroscience.2008.10.060

[32] Klingl YE, Pakravan D, Van Den Bosch L (2021) Opportunities for histone deacetylase inhibition in amyotrophic lateral sclerosis. Br J Pharmacol 178(6):1353–1372 32726472 10.1111/bph.15217 PMC9327724

[33] Alruwaili M, Al-Kuraishy HM, Alexiou A, Papadakis M, ALRashdi BM, Elhussieny O et al (2023) Pathogenic role of fibrinogen in the neuropathology of multiple sclerosis: a tale of sorrows and fears. Neurochem Res 48(11):3255–69 37442896 10.1007/s11064-023-03981-1 PMC10514123

[34] Al-Kuraishy HM, Jabir MS, Al-Gareeb AI, Saad HM, Batiha GE-S, Klionsky DJ (2023) The beneficial role of autophagy in multiple sclerosis: yes or no? Autophagy 31(4):435–44. 10.1007/s12264-015-1545-5 10.1007/s12264-015-1545-5 PMC10813579 37712858

[35] Al-Kuraishy HM, Al-Gareeb AI, Saad HM, Batiha GE-S (2023) The potential therapeutic effect of statins in multiple sclerosis: beneficial or detrimental effects. Inflammopharmacology 31(4):1671–1682. 10.1007/s10787-023-01240-x 37160526 10.1007/s10787-023-01240-x

[36] Booth D, Arthur A, Teutsch S, Bye C, Rubio J, Armati P et al (2005) Gene expression and genotyping studies implicate the interleukin 7 receptor in the pathogenesis of primary progressive multiple sclerosis. J Mol Med 83:822–830 16075257 10.1007/s00109-005-0684-y

[37] Ko R, Lee SY (2016) Glycogen synthase kinase 3β in Toll-like receptor signaling. BMB Rep 49(6):305 26996345 10.5483/BMBRep.2016.49.6.059 PMC5070717

[38] Ghosouri S, Soleimani M, Bakhtiari M, Ghasemi N (2023) Evaluation of in vivo lithium chloride effects as a GSK3-β inhibitor on human adipose derived stem cells differentiation into oligodendrocytes and re-myelination in an animal model of multiple sclerosis. Mol Biol Rep 50(2):1617–1625 36526850 10.1007/s11033-022-08181-8

[39] Pashaei S, Mohammadi P, Yarani R, Haghgoo SM, Aleagha MSE (2021) Carbohydrate and lipid metabolism in multiple sclerosis: clinical implications for etiology, pathogenesis, diagnosis, prognosis, and therapy. Arch Biochem Biophys 712:109030 34517010 10.1016/j.abb.2021.109030

[40] Gong L, Zhang QL, Zhang N, Hua WY, Huang YX, Di PW et al (2012) Neuroprotection by urate on 6-OHDA-lesioned rat model of Parkinson’s disease: linking to Akt/GSK 3β signaling pathway. J Neurochem 123(5):876–885 23094836 10.1111/jnc.12038

[41] Singh S, Mishra A, Bharti S, Tiwari V, Singh J, Parul et al (2018) Glycogen synthase kinase-3β regulates equilibrium between neurogenesis and gliogenesis in rat model of Parkinson’s disease: a crosstalk with Wnt and notch signaling. Mol Neurobiol 55:6500–6517 29327199 10.1007/s12035-017-0860-4

[42] Wang W, Yang Y, Ying C, Li W, Ruan H, Zhu X et al (2007) Inhibition of glycogen synthase kinase-3β protects dopaminergic neurons from MPTP toxicity. Neuropharmacology 52(8):1678–1684 17517424 10.1016/j.neuropharm.2007.03.017

[43] Khan SS, Janrao S, Srivastava S, Singh SB, Vora L, Khatri DK (2023) GSK-3β: an exuberating neuroinflammatory mediator in Parkinson’s disease. Biochem Pharmacol 210:115496. 10.1016/j.bcp.2023 36907495 10.1016/j.bcp.2023

[44] Cao Q, Karthikeyan A, Dheen ST, Kaur C, Ling E-A (2017) Production of proinflammatory mediators in activated microglia is synergistically regulated by Notch-1, glycogen synthase kinase (GSK-3β) and NF-κB/p65 signalling. PLoS ONE 12(10):e0186764 29049420 10.1371/journal.pone.0186764 PMC5648239

[45] Lei P, Ayton S, Bush AI, Adlard PA (2011) GSK-3 in neurodegenerative diseases. Int J Alzheimer’s Dis 2011:189246. 10.4061/2011/189246 21629738 10.4061/2011/189246 PMC3100544

[46] Li J, Ma S, Chen J, Hu K, Li Y, Zhang Z et al (2020) GSK-3β contributes to parkinsonian dopaminergic neuron death: evidence from conditional knockout mice and tideglusib. Front Mol Neurosci 13:81 32581704 10.3389/fnmol.2020.00081 PMC7283909

[47] Eriksson I (2022) Dealing with damaged lysosomes: impact of lysosomal membrane stability in health and disease: Linköping University Electronic Press

[48] Choi H-J, Cha SJ, Lee J-W, Kim H-J, Kim K (2020) Recent advances on the role of gsk3β in the pathogenesis of amyotrophic lateral sclerosis. Brain Sci 10(10):675 32993098 10.3390/brainsci10100675 PMC7600609

[49] Barr JL, Unterwald EM (2020) Glycogen synthase kinase-3 signaling in cellular and behavioral responses to psychostimulant drugs. Biochim et Biophys Acta (BBA)-Mol Cell Res 1867(9):118746 10.1016/j.bbamcr.2020.118746 PMC7313643 32454064

[50] Yang L, Zhang X, Li S, Wang H, Zhang X, Liu L et al (2020) Intranasal insulin ameliorates cognitive impairment in a rat model of Parkinson’s disease through Akt/GSK3β signaling pathway. Life Sci 259:118159 32763288 10.1016/j.lfs.2020.118159

[51] Kawakami F, Suzuki M, Shimada N, Kagiya G, Ohta E, Tamura K et al (2011) Stimulatory effect of α-synuclein on the tau-phosphorylation by GSK-3β. FEBS J 278(24):4895–4904 21985244 10.1111/j.1742-4658.2011.08389.x

[52] Wills J, Jones J, Haggerty T, Duka V, Joyce JN, Sidhu A (2010) Elevated tauopathy and alpha-synuclein pathology in postmortem Parkinson’s disease brains with and without dementia. Exp Neurol 225(1):210–218 20599975 10.1016/j.expneurol.2010.06.017 PMC2922478

[53] Duka T, Duka V, Joyce JN, Sidhu A (2009) α-Synuclein contributes to GSK-3β-catalyzed Tau phosphorylation in Parkinson’s disease models. FASEB J 23(9):2820 19369384 10.1096/fj.08-120410 PMC2796901

[54] Kwok JB, Hallupp M, Loy CT, Chan DK, Woo J, Mellick GD et al (2005) GSK3B polymorphisms alter transcription and splicing in Parkinson’s disease. Ann Neurol: Off J Am Neurol Assoc Child Neurol Soc 58(6):829–839 10.1002/ana.20691 16315267

[55] Takaichi Y, Chambers JK, Ano Y, Takashima A, Nakayama H, Uchida K (2021) Deposition of phosphorylated α-synuclein and activation of GSK-3β and PP2A in the PS19 mouse model of tauopathy. J Neuropathol Exp Neurol 80(8):731–740 34151989 10.1093/jnen/nlab054

[56] Van Kampen JM, Baranowski D, Kay DG (2014) Progranulin gene delivery protects dopaminergic neurons in a mouse model of Parkinson’s disease. PLoS ONE 9(5):e97032 24804730 10.1371/journal.pone.0097032 PMC4013129

[57] Gao X, Joselin AP, Wang L, Kar A, Ray P, Bateman A et al (2010) Progranulin promotes neurite outgrowth and neuronal differentiation by regulating GSK-3β. Protein Cell 1:552–562 21204008 10.1007/s13238-010-0067-1 PMC4875315

[58] Soni D, Kumar P (2022) GSK-3β-mediated regulation of Nrf2/HO-1 signaling as a new therapeutic approach in the treatment of movement disorders. Pharmacol Rep 74(4):557–569 35882765 10.1007/s43440-022-00390-z

[59] Di Martino RMC, Pruccoli L, Bisi A, Gobbi S, Rampa A, Martinez A et al (2020) Novel curcumin-diethyl fumarate hybrid as a dualistic GSK-3β inhibitor/Nrf2 inducer for the treatment of Parkinson’s disease. ACS Chem Neurosci 11(17):2728–2740 32663009 10.1021/acschemneuro.0c00363 PMC8009478

[60] Al-Kuraishy HM, Al-Kuraishi AH, Al-Windy S, Al-Gareeb AI (2019) Toxoplasmosis and risk of endothelial dysfunction: role of oxidative stress and pro-inflammatory mediators. Arch Clin Infect Dis 14(6). 10.5812/archcid.95563

[61] Al-Kuraishy HM, Al-Gareeb AI, Al-Maiahy TJ (2018) Concept and connotation of oxidative stress in preeclampsia. J Lab Phys 10(03):276–282 10.4103/JLP.JLP_26_18 PMC6052821 30078962

[62] Al-Hussaniy HA, Al-Kuraishy HM, Abdulameer A-GA (2022) The use of *Panax ginseng* to reduce the cardiotoxicity of doxorubicin and study its effect on modulating oxidative stress, inflammatory, and apoptosis pathways. Open Access Maced J Med Sci 10(A):715–9 10.3889/oamjms.2022.9479

[63] Al-Kuraishy HM, Al-Gareeb AI, Al-Niemi MS, Aljowaie RM, Almutairi SM, Alexiou A et al (2022) The prospective effect of allopurinol on the oxidative stress index and endothelial dysfunction in Covid-19. Inflammation 45(4):1651–1667 35199285 10.1007/s10753-022-01648-7 PMC8865950

[64] Blesa J, Trigo-Damas I, Quiroga-Varela A, Jackson-Lewis VR (2015) Oxidative stress and Parkinson’s disease. Front Neuroanat 9:91 26217195 10.3389/fnana.2015.00091 PMC4495335

[65] Ali NH, Alhamdan NA, Al-Kuraishy HM, Al-Gareeb AI, Elekhnawy E, Batiha GE-S (2023) Irisin/PGC-1α/FNDC5 pathway in Parkinson’s disease: truth under the throes. Naunyn-Schmiedeberg’s Arch Pharmacol 1–11. 10.1007/s00210-023-02726-9 10.1007/s00210-023-02726-9 37819389

[66] Gal S, Zheng H, Fridkin M, Youdim MB (2010) Restoration of nigrostriatal dopamine neurons in post-MPTP treatment by the novel multifunctional brain-permeable iron chelator-monoamine oxidase inhibitor drug, M30. Neurotox Res 17:15–27 19609632 10.1007/s12640-009-9070-9

[67] Al-Kuraishy HM, Jabir MS, Al-Gareeb AI, Albuhadily AK, Albukhaty S, Sulaiman GM et al (2023) Evaluation and targeting of amyloid precursor protein (APP)/amyloid beta (Aβ) axis in amyloidogenic and non-amyloidogenic pathways: a time outside the tunnel. Ageing Res Rev 92:102119. 10.1016/j.arr.2023.102119 37931848 10.1016/j.arr.2023.102119

[68] Blesa J, Przedborski S (2014) Parkinson’s disease: animal models and dopaminergic cell vulnerability. Front Neuroanat 8:155 25565980 10.3389/fnana.2014.00155 PMC4266040

[69] Khan Z, Ali SA (2018) Oxidative stress-related biomarkers in Parkinson’s disease: a systematic review and meta-analysis. Iran J Neurol 17(3):137 30886681 PMC6420691

[70] Rojo AI, de Sagarra MR, Cuadrado A (2008) GSK-3β down-regulates the transcription factor Nrf2 after oxidant damage: relevance to exposure of neuronal cells to oxidative stress. J Neurochem 105(1):192–202 18005231 10.1111/j.1471-4159.2007.05124.x

[71] Liu B, Zhang H, Tan X, Yang D, Lv Z, Jiang H et al (2017) GSPE reduces lead-induced oxidative stress by activating the Nrf2 pathway and suppressing miR153 and GSK-3β in rat kidney. Oncotarget 8(26):42226 28178683 10.18632/oncotarget.15033 PMC5522062

[72] Duarte AI, Santos P, Oliveira CR, Santos MS, Rego AC (2008) Insulin neuroprotection against oxidative stress is mediated by Akt and GSK-3β signaling pathways and changes in protein expression. Biochim et Biophys Acta (BBA)-Mol Cell Res 1783(6):994–1002 10.1016/j.bbamcr.2008.02.016 18348871

[73] Wang D, Yang Y, Zou X, Zheng Z, Zhang J (2020) Curcumin ameliorates CKD-induced mitochondrial dysfunction and oxidative stress through inhibiting GSK-3β activity. J Nutr Biochem 83:108404 32531667 10.1016/j.jnutbio.2020.108404

[74] Al-Kuraishy HM, Al-Gareeb AI, Fageyinbo MS, Batiha GE-S (2022) Vinpocetine is the forthcoming adjuvant agent in the management of COVID-19. Futur Sci OA 8(5):FSO797. 10.2144/fsoa-2021-0099 10.2144/fsoa-2021-0099 PMC9017700 35662743

[75] Dolatshahi M, Ranjbar Hameghavandi MH, Sabahi M, Rostamkhani S (2021) Nuclear factor-kappa B (NF-κB) in pathophysiology of Parkinson disease: diverse patterns and mechanisms contributing to neurodegeneration. Eur J Neurosci 54(1):4101–4123 10.1111/ejn.15242 33884689

[76] Buss H, Dorrie A, Schmitz ML, Frank R, Livingstone M, Resch K et al (2004) Phosphorylation of serine 468 by GSK-3β negatively regulates basal p65 NF-κB activity. J Biol Chem 279(48):49571–49574 15465828 10.1074/jbc.C400442200

[77] Chen H, Yang S, Yang Z, Ma L, Jiang D, Mao J et al (2007) Inhibition of GSK-3β decreases NF-κB-dependent gene expression and impairs the rat liver regeneration. J Cell Biochem 102(5):1281–1289 17427960 10.1002/jcb.21358

[78] Batiha GE-S, Al-Gareeb AI, Rotimi D, Adeyemi OS, Al-Kuraishy HM (2022) Common NLRP3 inflammasome inhibitors and Covid-19: divide and conquer. Sci Afr 18:e01407 36310607 10.1016/j.sciaf.2022.e01407 PMC9595499

[79] de Araújo FM, Cuenca-Bermejo L, Fernandez-Villalba E, Costa SL, Silva VDA, Herrero MT (2022) Role of microgliosis and NLRP3 inflammasome in Parkinson’s disease pathogenesis and therapy. Cell Mol Neurobiol 42(5):1283–1300 33387119 10.1007/s10571-020-01027-6 PMC11421755

[80] Wang S, Yuan Y-H, Chen N-H, Wang H-B (2019) The mechanisms of NLRP3 inflammasome/pyroptosis activation and their role in Parkinson’s disease. Int Immunopharmacol 67:458–464 30594776 10.1016/j.intimp.2018.12.019

[81] Fan Z, Pan Y-T, Zhang Z-Y, Yang H, Yu S-Y, Zheng Y et al (2020) Systemic activation of NLRP3 inflammasome and plasma α-synuclein levels are correlated with motor severity and progression in Parkinson’s disease. J Neuroinflammation 17(1):1–10 31915018 10.1186/s12974-019-1670-6 PMC6950934

[82] Wang S-H, Cui L-G, Su X-L, Komal S, Ni R-C, Zang M-X et al (2022) GSK-3β-mediated activation of NLRP3 inflammasome leads to pyroptosis and apoptosis of rat cardiomyocytes and fibroblasts. Eur J Pharmacol 920:174830 35182545 10.1016/j.ejphar.2022.174830

[83] Zhu P, Zhang J-J, Cen Y, Yang Y, Wang F, Gu K-P et al (2022) High endogenously synthesized N-3 polyunsaturated fatty acids in Fat-1 mice attenuate high-fat diet-induced insulin resistance by inhibiting NLRP3 inflammasome activation via Akt/GSK-3β/TXNIP pathway. Molecules 27(19):6384 36234919 10.3390/molecules27196384 PMC9570616

[84] Batiha GE-S, Al-Kuraishy HM, Al-Gareeb AI, Alruwaili M, AlRuwaili R, Albogami SM et al (2023) Targeting of neuroinflammation by glibenclamide in Covid-19: old weapon from arsenal. Inflammopharmacology 31(1):1–7 36418600 10.1007/s10787-022-01087-8 PMC9685016

[85] Al-Kuraishy HM, Al-Gareeb AI, Rauf A, Alhumaydhi FA, Kujawska M, El-Saber Batiha G (2023) Mechanistic insight and possible mechanism of seizure in Covid-19: the nuances and focal points. CNS Neurol Disord-Drug Targets (Formerly Current Drug Targets-CNS & Neurological Disorders) 22(6):875–83 10.2174/1871527321666220517115227 35585806

[86] Alkhayyat SS, Al-Kuraishy HM, Al-Gareeb AI, El-Bouseary MM, AboKamer AM, Batiha GE-S et al (2022) Fenofibrate for COVID-19 and related complications as an approach to improve treatment outcomes: the missed key for Holy Grail. Inflamm Res 71(10–11):1159–1167 35941297 10.1007/s00011-022-01615-w PMC9360649

[87] Hirsch EC, Vyas S, Hunot S (2012) Neuroinflammation in Parkinson’s disease. Parkinsonism Relat Disord 18:S210–S212 22166438 10.1016/S1353-8020(11)70065-7

[88] Tiwari PC, Pal R (2022) The potential role of neuroinflammation and transcription factors in Parkinson disease. Dialogues Clin Neurosci 19(1):71–80. 10.31887/DCNS.2017.19.1/rpal 10.31887/DCNS.2017.19.1/rpal PMC5442366 28566949

[89] Reynolds AD, Stone DK, Hutter JA, Benner EJ, Mosley RL, Gendelman HE (2010) Regulatory T cells attenuate Th17 cell-mediated nigrostriatal dopaminergic neurodegeneration in a model of Parkinson’s disease. J Immunol 184(5):2261–2271 20118279 10.4049/jimmunol.0901852 PMC2824790

[90] Clark LF, Kodadek T (2016) The immune system and neuroinflammation as potential sources of blood-based biomarkers for Alzheimer’s disease, Parkinson’s disease, and Huntington’s disease. ACS Chem Neurosci 7(5):520–527 27046268 10.1021/acschemneuro.6b00042

[91] Orellana AMM, Vasconcelos AR, Leite JA, de Sá LL, Andreotti DZ, Munhoz CD et al (2015) Age-related neuroinflammation and changes in AKT-GSK-3β and WNT/β-CATENIN signaling in rat hippocampus. Aging (Albany NY) 7(12):1094 26647069 10.18632/aging.100853 PMC4712335

[92] Wang X, Chen L, Xu Y, Wang W, Wang Y, Zhang Z et al (2021) Gastrodin alleviates perioperative neurocognitive dysfunction of aged mice by suppressing neuroinflammation. Eur J Pharmacol 892:173734 33220272 10.1016/j.ejphar.2020.173734

[93] Arab HH, Safar MM, Shahin NN (2021) Targeting ROS-dependent AKT/GSK-3β/NF-κB and DJ-1/Nrf2 pathways by dapagliflozin attenuates neuronal injury and motor dysfunction in rotenone-induced Parkinson’s disease rat model. ACS Chem Neurosci 12(4):689–703 33543924 10.1021/acschemneuro.0c00722

[94] Lee S, Hong DG, Yang S, Kim J, Baek M, Kim S et al (2022) Anti-inflammatory effect of IKK-activated GSK-3β inhibitory peptide prevented nigrostriatal neurodegeneration in the rodent model of Parkinson’s disease. Int J Mol Sci 23(2):998 35055183 10.3390/ijms23020998 PMC8779943

[95] Gao L, Zhang Y, Sterling K, Song W (2022) Brain-derived neurotrophic factor in Alzheimer’s disease and its pharmaceutical potential. Transl Neurodegener 11(1):1–34 35090576 10.1186/s40035-022-00279-0 PMC8796548

[96] Ali NH, Al-Kuraishy HM, Al-Gareeb AI, Alnaaim SA, Alexiou A, Papadakis M et al (2023) The probable role of tissue plasminogen activator/neuroserpin axis in Alzheimer’s disease: a new perspective. Acta Neurol Belg 1–12. 10.1007/s13760-023-02403-x 10.1007/s13760-023-02403-x PMC10965687 37917293

[97] Alnaaim SA, Al-kuraishy HM, Al-Gareeb AI, Ali NH, Alexiou A, Papadakis M et al (2023) New insights on the potential anti-epileptic effect of metformin: mechanistic pathway. J Cell Mol Med 27(24):3953–3965. 10.1111/jcmm.17965 37737447 10.1111/jcmm.17965 PMC10747420

[98] Ali NH, Al‐Kuraishy HM, Al‐Gareeb AI, Albuhadily AK, Hamad RS, Alexiou A et al (2023) Role of brain renin–angiotensin system in depression: a new perspective. CNS Neurosci Ther. 10.1111/cns.14525 10.1111/cns.14525 PMC11017442 37953501

[99] Rahmani F, Saghazadeh A, Rahmani M, Teixeira AL, Rezaei N, Aghamollaii V et al (2019) Plasma levels of brain-derived neurotrophic factor in patients with Parkinson disease: a systematic review and meta-analysis. Brain Res 1704:127–136 30296429 10.1016/j.brainres.2018.10.006

[100] Chang E, Wang J (2021) Brain-derived neurotrophic factor attenuates cognitive impairment and motor deficits in a mouse model of Parkinson’s disease. Brain Behav 11(8):e2251 34132500 10.1002/brb3.2251 PMC8413743

[101] Ricci V, Pomponi M, Martinotti G, Bentivoglio A, Loria G, Bernardini S et al (2010) Antidepressant treatment restores brain-derived neurotrophic factor serum levels and ameliorates motor function in Parkinson disease patients. J Clin Psychopharmacol 30(6):751–753 21057246 10.1097/JCP.0b013e3181fc2ec7

[102] Li X-T, Liang Z, Wang T-T, Yang J-W, Ma W, Deng S-K et al (2017) Brain-derived neurotrophic factor promotes growth of neurons and neural stem cells possibly by triggering the phosphoinositide 3-kinase/AKT/glycogen synthase kinase-3β/β-catenin pathway. CNS Neurol Disord-Drug Targets (Formerly Current Drug Targets-CNS & Neurological Disorders) 16(7):828–36 10.2174/1871527316666170518170422 28524001

[103] Xia Y, Wang CZ, Liu J, Anastasio NC, Johnson KM (2010) Brain-derived neurotrophic factor prevents phencyclidine-induced apoptosis in developing brain by parallel activation of both the ERK and PI-3K/Akt pathways. Neuropharmacology 58(2):330–336 19887077 10.1016/j.neuropharm.2009.10.009 PMC2813345

[104] Wada A (2009) Lithium and neuropsychiatric therapeutics: neuroplasticity via glycogen synthase kinase-3β, β-catenin, and neurotrophin cascades. J Pharmacol Sci 110(1):14–28 19423950 10.1254/jphs.09R02CR

[105] Ullah A, Ali N, Ahmad S, Rahman S, Alghamdi S, Bannunah A et al (2021) Glycogen synthase kinase-3 (GSK-3) a magic enzyme: it’s role in diabetes mellitus and glucose homeostasis, interactions with fluroquionlones. A mini-review, Braz J Biol, p 83 10.1590/1519-6984.250179 34524376

[106] Arciniegas Ruiz SM, Eldar-Finkelman H (2022) Glycogen synthase kinase-3 inhibitors: preclinical and clinical focus on CNS-A decade onward. Front Mol Neurosci 14:792364 35126052 10.3389/fnmol.2021.792364 PMC8813766

[107] Martínez-González L, Gonzalo-Consuegra C, Gómez-Almería M, Porras G, de Lago E, Martín-Requero Á et al (2021) Tideglusib, a non-ATP competitive inhibitor of GSK-3β as a drug candidate for the treatment of amyotrophic lateral sclerosis. Int J Mol Sci 22(16):8975 34445680 10.3390/ijms22168975 PMC8396476

[108] Bhat RV, Andersson U, Andersson S, Knerr L, Bauer U, Sundgren-Andersson AK (2018) The conundrum of GSK3 inhibitors: is it the dawn of a new beginning? J Alzheimers Dis 64(s1):S547–S554 29758944 10.3233/JAD-179934

[109] Pandey MK, DeGrado TR (2016) Glycogen synthase kinase-3 (GSK-3)-targeted therapy and imaging. Theranostics 6(4):571 26941849 10.7150/thno.14334 PMC4775866

[110] Bastide MF, Bido S, Duteil N, Bézard E (2016) Striatal NELF-mediated RNA polymerase II stalling controls l-dopa induced dyskinesia. Neurobiol Dis 85:93–98 26480869 10.1016/j.nbd.2015.10.013

[111] Kumar A, Srivastava G, Negi AS, Sharma A (2019) Docking, molecular dynamics, binding energy-MM-PBSA studies of naphthofuran derivatives to identify potential dual inhibitors against BACE-1 and GSK-3β. J Biomol Struct Dyn 37(2):275–290 29310523 10.1080/07391102.2018.1426043

[112] Wei J, Wang J, Zhang J, Yang J, Wang G, Wang Y (2022) Development of inhibitors targeting glycogen synthase kinase-3β for human diseases: strategies to improve selectivity. Eur J Med Chem 236:114301 35390715 10.1016/j.ejmech.2022.114301

[113] Yao M, Teng H, Lv Q, Gao H, Guo T, Lin Y et al (2021) Anti-hyperglycemic effects of dihydromyricetin in streptozotocin-induced diabetic rats. Food Sci Human Wellness 10(2):155–162 10.1016/j.fshw.2021.02.004

[114] Snitow ME, Bhansali RS, Klein PS (2021) Lithium and therapeutic targeting of GSK-3. Cells 10(2):255 33525562 10.3390/cells10020255 PMC7910927

[115] Shri SR, Manandhar S, Nayak Y, Pai KSR (2023) Role of GSK-3β inhibitors: new promises and opportunities for Alzheimer’s disease. Adv Pharm Bull 13(4):688 38022801 10.34172/apb.2023.071 PMC10676556

[116] Mumtaz I, Ayaz MO, Khan MS, Manzoor U, Ganayee MA, Bhat AQ et al (2022) Clinical relevance of biomarkers, new therapeutic approaches, and role of post-translational modifications in the pathogenesis of Alzheimer’s disease. Front Aging Neurosci 14:977411 36158539 10.3389/fnagi.2022.977411 PMC9490081

[117] Eldar-Finkelman H, Martinez A (2011) GSK-3 inhibitors: preclinical and clinical focus on CNS. Front Mol Neurosci 4:32 22065134 10.3389/fnmol.2011.00032 PMC3204427

[118] Jeffers A, Qin W, Owens S, Koenig KB, Komatsu S, Giles FJ et al (2019) Glycogen synthase kinase-3β inhibition with 9-ING-41 attenuates the progression of pulmonary fibrosis. Sci Rep 9(1):18925 31831767 10.1038/s41598-019-55176-w PMC6908609

[119] Nam G-H, Jo K-J, Park Y-S, Kawk HW, Yoo J-G, Jang JD et al (2019) *Bacillus*/*Trapa japonica* Fruit Extract Ferment Filtrate enhances human hair follicle dermal papilla cell proliferation via the Akt/ERK/GSK-3β signaling pathway. BMC Complement Altern Med 19(1):1–11 31088549 10.1186/s12906-019-2514-8 PMC6518747

[120] Wu Q, Ma J, Wei J, Meng W, Wang Y, Shi M (2021) lncRNA SNHG11 promotes gastric cancer progression by activating the Wnt/β-catenin pathway and oncogenic autophagy. Mol Ther 29(3):1258–1278 33068778 10.1016/j.ymthe.2020.10.011 PMC7934455

[121] Li R, Liu Z, Wu X, Yu Z, Zhao S, Tang X (2019) Lithium chloride promoted hematoma resolution after intracerebral hemorrhage through GSK-3β-mediated pathways-dependent microglia phagocytosis and M2-phenotype differentiation, angiogenesis and neurogenesis in a rat model. Brain Res Bull 152:117–127 31325596 10.1016/j.brainresbull.2019.07.019

[122] Parkin GM, Thomas EA (2022) Provider perspectives on the current use of lithium medications and lithium monitoring practices for psychiatric conditions. Neuropsychiatr Dis Treat 2083–93 10.2147/NDT.S377261 PMC9484562 36133030

[123] Ataallah B, Al-Zakhari R, Sharma A, Tofano M, Haggerty G (2020) A rare but reversible cause of lithium-induced bradycardia. Cureus 12(6):e8600. 10.7759/cureus.8600 32676239 10.7759/cureus.8600 PMC7362593

[124] Czarnywojtek A, Zgorzalewicz-Stachowiak M, Czarnocka B, Sawicka-Gutaj N, Gut P, Krela-Kazmierczak I et al (2020) Effect of lithium carbonate on the function of the thyroid gland: mechanism of action and clinical implications. J Physiol Pharmacol 71(2). 10.26402/jpp.2020.2.03 10.26402/jpp.2020.2.03 32633237

[125] Shalaby HN, Zaki HF, Ain-Shoka AAA, Mohammed RA (2022) Adenosine A2A receptor blockade ameliorates mania like symptoms in rats: signaling to PKC-α and Akt/GSK-3β/β-catenin. Mol Neurobiol 59(10):6397–6410 35943710 10.1007/s12035-022-02977-2 PMC9463338

[126] Engel T, Goñi-Oliver P, Lucas JJ, Avila J, Hernández F (2006) Chronic lithium administration to FTDP-17 tau and GSK-3β overexpressing mice prevents tau hyperphosphorylation and neurofibrillary tangle formation, but pre-formed neurofibrillary tangles do not revert. J Neurochem 99(6):1445–1455 17059563 10.1111/j.1471-4159.2006.04139.x

[127] Lazzara CA, Kim Y-H (2015) Potential application of lithium in Parkinson’s and other neurodegenerative diseases. Front Neurosci 9:403 26578864 10.3389/fnins.2015.00403 PMC4621308

[128] Guttuso T Jr, Shepherd R, Frick L, Feltri ML, Frerichs V, Ramanathan M et al (2023) Lithium’s effects on therapeutic targets and MRI biomarkers in Parkinson’s disease: a pilot clinical trial. IBRO Neurosci Rep 14:429–434 37215748 10.1016/j.ibneur.2023.05.001 PMC10196787

[129] Yang H, George SJ, Thompson DA, Silverman HA, Tsaava T, Tynan A et al (2022) Famotidine activates the vagus nerve inflammatory reflex to attenuate cytokine storm. Mol Med 28(1):1–13 35578169 10.1186/s10020-022-00483-8 PMC9109205

[130] Angeli A, Pinteala M, Maier SS, Del Prete S, Capasso C, Simionescu BC et al (2019) Inhibition of α-, β-, γ-, δ-, ζ- and η-class carbonic anhydrases from bacteria, fungi, algae, diatoms and protozoans with famotidine. J Enzyme Inhib Med Chem 34(1):644–650 30727781 10.1080/14756366.2019.1571273 PMC6366436

[131] Unal G, Dokumaci AH, Ozkartal CS, Yerer MB, Aricioglu F (2019) Famotidine has a neuroprotective effect on MK-801 induced toxicity via the Akt/GSK-3β/β-catenin signaling pathway in the SH-SY5Y cell line. Chem Biol Interact 314:108823 31563592 10.1016/j.cbi.2019.108823

[132] Unal G, Aricioglu F (2020) Famotidine improved schizophrenia-like behaviors in acute ketamine model of schizophrenia in rats. Psychiatry Behav Sci 10(2):45 10.5455/PBS.20200330095749

[133] Molinari S, Kaminski R, Di Rocco A, Yahr M (1995) The use of famotidine in the treatment of Parkinson’s disease: a pilot study. J Neural Transm-Parkinson’s Dis Dement Sect 9:243–247 10.1007/BF02259665 8527008

[134] Huot P, Fox SH (2011) Nondopaminergic treatments for Parkinson’s disease. Neurodegener Dis Manag 1(6):491–512 10.2217/nmt.11.62 PMC4976881 27230697

[135] Mestre TA, Shah BB, Connolly BS, de Aquino C, Al Dhakeel A, Walsh R et al (2014) Famotidine, a histamine H2 receptor antagonist, does not reduce levodopa-induced dyskinesia in Parkinson’s disease: a proof-of-concept study. Mov Disord Clin Pract 1(3):219–224 30363717 10.1002/mdc3.12061 PMC6182979

[136] Ríos ALM, Gutierrez-Suarez K, Carmona Z, Ramos CG, Oliveira LFS (2022) Pharmaceuticals as emerging pollutants: case naproxen an overview. Chemosphere 291:132822 34767851 10.1016/j.chemosphere.2021.132822

[137] Motawi TM, Bustanji Y, EL-Maraghy SA, Taha MO, Al Ghussein MA (2013) Naproxen and cromolyn as new glycogen synthase kinase 3β inhibitors for amelioration of diabetes and obesity: an investigation by docking simulation and subsequent in vitro/in vivo biochemical evaluation. J Biochem Mol Toxicol 27(9):425–36 23784744 10.1002/jbt.21503

[138] Motawi TM, Bustanji Y, El-Maraghy S, Taha MO, Al-Ghussein MA (2014) Evaluation of naproxen and cromolyn activities against cancer cells viability, proliferation, apoptosis, p53 and gene expression of survivin and caspase-3. J Enzyme Inhib Med Chem 29(2):153–161 23368763 10.3109/14756366.2012.762645

[139] Greenspan EJ, Madigan JP, Boardman LA, Rosenberg DW (2011) Ibuprofen inhibits activation of nuclear β-catenin in human colon adenomas and induces the phosphorylation of GSK-3β. Cancer Prev Res 4(1):161–171 10.1158/1940-6207.CAPR-10-0021 PMC3078769 21205744

[140] Singh A, Tripathi P, Singh S (2021) Neuroinflammatory responses in Parkinson’s disease: relevance of Ibuprofen in therapeutics. Inflammopharmacology 29:5–14 33052479 10.1007/s10787-020-00764-w

[141] Al-kuraishy HM, Al-Gareeb AI, Alexiou A, Papadakis M, Nadwa EH, Albogami SM et al (2022) Metformin and growth differentiation factor 15 (GDF15) in type 2 diabetes mellitus: a hidden treasure. J Diabetes 14(12):806–814. 10.1111/1753-0407 36444166 10.1111/1753-0407 PMC9789395

[142] Alrouji M, Al-Kuraishy HM, Al-Gareeb AI, Ashour NA, Jabir MS, Negm WA et al (2023) Metformin role in Parkinson’s disease: a double-sword effect. Mol Cell Biochem 1–17. 10.1007/s11010-023-04771-7 10.1007/s11010-023-04771-7 37266747

[143] Agostini F, Masato A, Bubacco L, Bisaglia M (2021) Metformin repurposing for Parkinson disease therapy: opportunities and challenges. Int J Mol Sci 23(1):398 35008822 10.3390/ijms23010398 PMC8745385

[144] Luo Z, Zhu T, Luo W, Lv Y, Zhang L, Wang C et al (2019) Metformin induces apoptotic cytotoxicity depending on AMPK/PKA/GSK-3β-mediated c-FLIPL degradation in non-small cell lung cancer. Cancer Manag Res 11:681–689. 10.2147/CMAR.S178688 30666163 10.2147/CMAR.S178688 PMC6331071

[145] Cao F, Yang K, Qiu S, Li J, Jiang W, Tao L et al (2023) Metformin reverses oxidative stress-induced mitochondrial dysfunction in pre-osteoblasts via the EGFR/GSK-3β/calcium pathway. Int J Mol Med 51(4):1–13 36999607 10.3892/ijmm.2023.5239 PMC10049025

[146] Al-kuraishy HM, Al-Gareeb AI, Alexiou A, Papadakis M, Nadwa EH, Albogami SM et al (2022) Metformin and growth differentiation factor 15 (GDF15) in type 2 diabetes mellitus: a hidden treasure. J Diabetes 14(12):806–814 36444166 10.1111/1753-0407.13334 PMC9789395

[147] Su J, Zhang J, Bao R, Xia C, Zhang Y, Zhu Z et al (2021) Mitochondrial dysfunction and apoptosis are attenuated through activation of AMPK/GSK-3β/PP2A pathway in Parkinson’s disease. Eur J Pharmacol 907:174202 34048739 10.1016/j.ejphar.2021.174202

[148] Ren Z-X, Zhao Y-F, Cao T, Zhen X-C (2016) Dihydromyricetin protects neurons in an MPTP-induced model of Parkinson’s disease by suppressing glycogen synthase kinase-3 beta activity. Acta Pharmacol Sin 37(10):1315–1324 27374489 10.1038/aps.2016.42 PMC5057232

[149] Morales-García JA, Susín C, Alonso-Gil S, Pérez DI, Palomo V, Pérez C et al (2013) Glycogen synthase kinase-3 inhibitors as potent therapeutic agents for the treatment of Parkinson disease. ACS Chem Neurosci 4(2):350–360 23421686 10.1021/cn300182g PMC3582296

[150] Armagan G, Sevgili E, Gürkan FT, Köse FA, Bilgiç T, Dagcı T et al (2019) Regulation of the Nrf2 pathway by glycogen synthase kinase-3β in MPP^+^-induced cell damage. Molecules 24(7):1377 30965670 10.3390/molecules24071377 PMC6480928

[151] Yuskaitis CJ, Jope RS (2009) Glycogen synthase kinase-3 regulates microglial migration, inflammation, and inflammation-induced neurotoxicity. Cell Signal 21(2):264–273 19007880 10.1016/j.cellsig.2008.10.014 PMC2630396

[152] Del Ser T, Steinwachs KC, Gertz HJ, Andres MV, Gomez-Carrillo B, Medina M et al (2013) Treatment of Alzheimer’s disease with the GSK-3 inhibitor tideglusib: a pilot study. J Alzheimers Dis 33(1):205–215 22936007 10.3233/JAD-2012-120805

[153] Höglinger GU, Huppertz HJ, Wagenpfeil S, Andrés MV, Belloch V, León T et al (2014) Tideglusib reduces progression of brain atrophy in progressive supranuclear palsy in a randomized trial. Mov Disord 29(4):479–487 24488721 10.1002/mds.25815

[154] Tolosa E, Litvan I, Höglinger GU, Burn D, Lees A, Andrés MV et al (2014) A phase 2 trial of the GSK-3 inhibitor tideglusib in progressive supranuclear palsy. Mov Disord 29(4):470–478 24532007 10.1002/mds.25824

[155] Lovestone S, Boada M, Dubois B, Hüll M, Rinne JO, Huppertz H-J et al (2015) A phase II trial of tideglusib in Alzheimer’s disease. J Alzheimers Dis 45(1):75–88 25537011 10.3233/JAD-141959

[156] Anagnostou E, Bennett TA, Thorpe K, Nicolson R (2018) A phase 2 randomized, placebo-controlled trial of tideglusib, an orally administered GSK-3 beta inhibitor, in the treatment of adolescents with ASD. 65th Annual Meeting AACAP

[157] Horrigan J, McMorn A, Snape M, Nikolenko N, Gomes T, Lochmuller H (2018) AMO-02 (tideglusib) for the treatment of congenital and childhood onset myotonic dystrophy type 1. Neuromuscul Disord 28:S14 10.1016/S0960-8966(18)30330-4 32942085

[158] Lazzara CA, Riley RR, Rane A, Andersen JK, Kim Y-H (2015) The combination of lithium and l-Dopa/Carbidopa reduces MPTP-induced abnormal involuntary movements (AIMs) via calpain-1 inhibition in a mouse model: relevance for Parkinson’s disease therapy. Brain Res 1622:127–136 26119916 10.1016/j.brainres.2015.06.018 PMC4562891

[159] Marras C, Herrmann N, Fischer HD, Fung K, Gruneir A, Rochon PA et al (2016) Lithium use in older adults is associated with increased prescribing of parkinson medications. Am J Geriatr Psychiatry 24(4):301–309 27037047 10.1016/j.jagp.2015.11.004

[160] Zhang J, Anshul F, Malhotra DK, Jaume J, Dworkin LD, Gong R (2021) Microdose lithium protects against pancreatic islet destruction and renal impairment in streptozotocin-elicited diabetes. Antioxidants 10(1):138 33478120 10.3390/antiox10010138 PMC7835906

